# Predictors of Frequency and Success of Wild Meat Hunting Trips and Carcass Prices in an African Biodiversity Hotspot

**DOI:** 10.1007/s10745-025-00572-2

**Published:** 2025-01-25

**Authors:** Charles A. Emogor, Daniel J. Ingram, Andrew Balmford, Robert J. Fletcher, Diane Detoeuf, Ben Balmford, Dan O. Agbor, Lauren Coad

**Affiliations:** 1 Conservation Science Group, Department of Zoology, University of Cambridge, Cambridge, CB2 3EJ UK; 2 Pangolin Protection Network, Calabar, Nigeria; 3 Wildlife Conservation Society, 2300 Southern Boulevard Bronx, New York, NY USA; 4 Durrell Institute of Conservation and Ecology (DICE), School of Natural Sciences, University of Kent, Canterbury, CT2 7NR UK; 5 Department of Wildlife Ecology and Conservation, University of Florida, Gainesville, USA; 6 Economics Department, Land, Environment, Economics and Policy Institute, University of Exeter, Devon, UK; 7 Center for International Forestry Research, Bogor, Indonesia; 8 Interdisciplinary Centre for Conservation Science, University of Oxford, Oxford, UK

**Keywords:** Hunter behavior, Wild meat, Hunter offtake, Biological resource use, Cross River National Park, Nigeria, West Africa

## Abstract

Hunting wild animals for food and income, which is pervasive across tropical regions, drives biodiversity loss. Interventions to promote sustainable wild meat harvesting require information on hunter behavior. Here we monitored the hunting activities of 33 hunters in SE Nigeria over three years (1,106 hunter-months) to identify correlates of (a) the probability of initiating a hunting trip on any given day; (b) trip success – whether an animal was caught, and if so, how many; and (c) carcass price. We found a higher probability of initiating a trip during periods with bright moon phases and in peak agriculture season. Hunters were more likely to catch at least one animal when there was less rainfall and on shorter hunting trips. However, among successful trips, the number of animals caught increased with trip duration. Taken together, these results suggest hunters set themselves a minimum target of not returning empty-handed rather than optimally adjusting their hunting effort. Lastly, the carcass price per kilogram of a species’ meat increased with its palatability but decreased with mass, with the fall in price observed to be greater for rarely caught, smaller-bodied animals than more frequently caught animals. Our results provide deeper insights into the behavioral plasticity of wild meat hunters.

**Supplementary Information:**

The online version contains supplementary material available at 10.1007/s10745-025-00572-2.

## Introduction

Hunting wild animals and the trade and consumption of their meat (hereafter wild meat) have high cultural and socio-economic significance across tropical regions (Ingram et al., 2021). Wild meat provides food and income for local economies (Coad et al., 2019; Friant et al., 2020) and is used extensively in traditional medicines and festivities (Friant et al., 2022). It is an important source of protein in areas where domestic alternatives are scarce and expensive (Fa et al., 2015; Torres et al., 2022). Furthermore, in West and Central Africa alone, thousands of tons of wild meat are traded annually (Cowlishaw et al., 2005; Fa et al., 2006), generating an estimated $42–206 million per year (Davies, 2002; Lescuyer & Nasi, 2016).

Nonetheless, current offtake levels are potentially unsustainable, adversely affecting ecosystem functioning and possibly negatively impacting human well-being and livelihoods (Abernethy et al., 2013; Bello et al., 2015). Promoting the sustainability of wild meat harvesting requires information on offtake patterns and hunter behavior, which can inform suitable interventions and improve the robustness of offtake sustainability indicators (Coad et al., 2019; Riddell et al., 2022). Earlier work has documented substantial flexibility in hunter behavior, including a change to using LED (light-emitting diode) flashlights to increase the efficiency of nocturnal hunting (Bowler et al., 2020), higher levels of offtake during COVID-19 lockdown to buffer the socioeconomic impacts of the lockdown (Emogor et al., 2024a), and targeting bigger prey following an increased tax on shotgun cartridges (Sirén & Wilkie, 2016). These adaptations suggest that hunter behavior is not fixed, and that greater understanding of the dynamics of hunter decision-making may be helpful.

Here we use an exceptionally large longitudinal hunting dataset that we assembled by following 33 hunters continuously for three years (1,106 hunter-months) to ask three questions: What factors predict the decision to undertake a hunting trip? What are the correlates of the success of hunting trips? And lastly, what are the predictors of the price of hunted carcasses? In answering the first and second questions, we examine the associations between socio-economic (e.g., food requirements and income) and biophysical factors (e.g., rainfall and moon phase) and the probability of initiating a trip on any given day, the probability that at least one animal is captured on a trip, and the number of animals caught on trips that captured at least one animal. For the last question, we explored the relationships between socio-cultural (e.g., meat palatability) and biological characteristics (e.g., mass) and carcass price, aiming to identify species with higher market values that might therefore be preferentially hunted.

## Methods

### Study Location and Data Collection

**Fig. 1:**
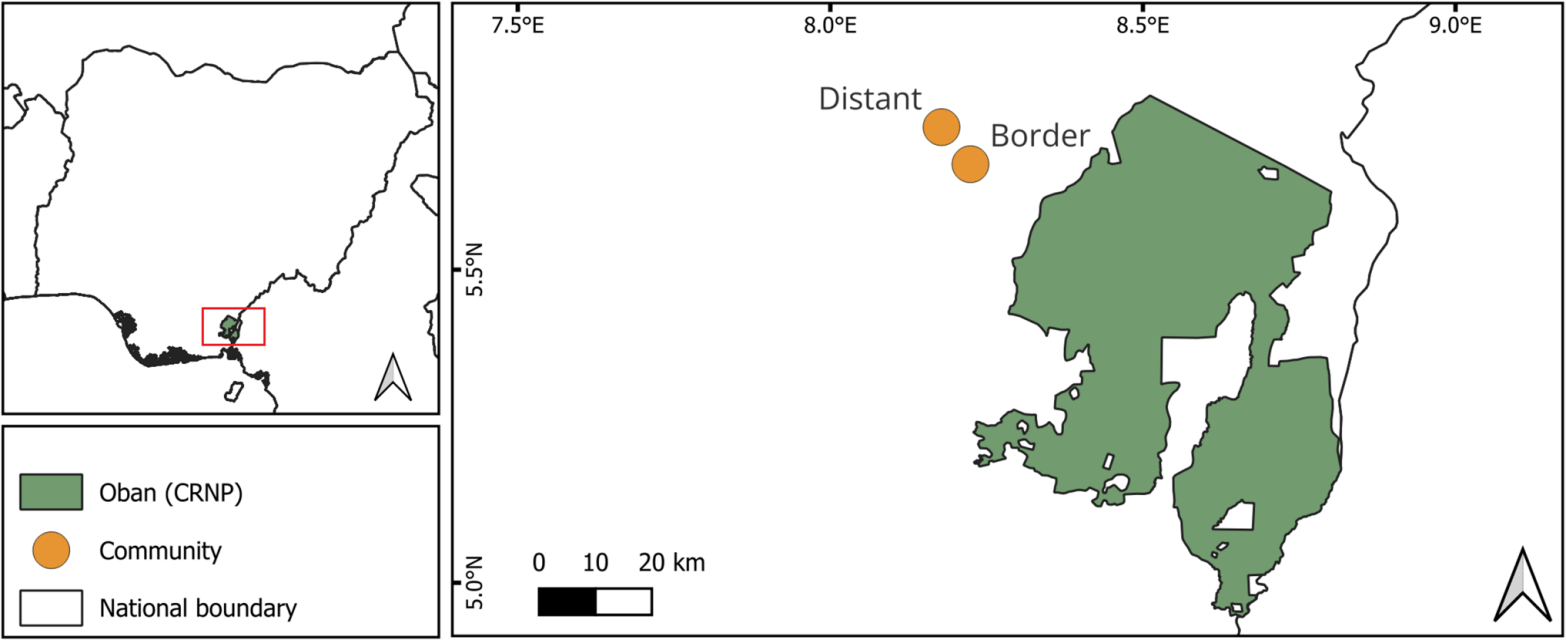
Approximate locations of the study communities around Oban Division of Nigeria’s Cross River National Park. The red rectangle in the top left map highlights the study location within Nigeria

We continuously monitored the hunting activities of 33 male hunters in two communities near Oban Division of Nigeria’s Cross River National Park (CRNP; Fig. 1) over three years (April 2020-March 2023). The park, located in a global biodiversity hotspot (Myers et al., 2000), comprises two divisions. Oban, the southern division is ~3,000 km² of lowland forest and adjacent to Korup National Park in Cameroon, while Okwangwo, 65 km north of Oban, covers 1,000 km² of montane forest and is contiguous with Cameroon’s Takamanda National Park. The area has distinct wet and dry seasons from April-October and November-March, respectively (Akpan & Offem, 1993). To retain anonymity, we refer to the communities based on their proximity to the park: border (~ 10 km from park boundary; 19 hunters) and distant (~ 20 km from park boundary; 14 hunters).

We consulted each community’s hunter association to recruit hunters for the study. We first provided information about our research, including the data collection protocol before assigning unique identifiers to hunters who volunteered to take part. Fourteen and nine male hunters in the border and distant communities, respectively, who were present in the initial consultation did not volunteer. While our sample of hunters is thus clearly self-selected, we note that it likely captures the breadth of hunter characteristics within these communities (for example, their age ranged from 26 to 76 years). Importantly, we did not follow casual hunters (who predominantly hunt with snares) as they are not members of hunter associations – we thus focused entirely on formal hunters (who mainly use guns). This exclusion is largely because trapping requires relatively minimal skills and can be practiced by anyone with access to wire snares.

Using a questionnaire adapted from WILDMEAT project (https://www.wildmeat.org/), field researchers recruited from the focal community interviewed hunters at the end of each hunting trip they made, recording trip duration (in days) and the number of animals caught. For each animal caught, we recorded hunting method(s), age (adult or juvenile), sex (where known), intended use (household consumption, ceremonial use, or commercial), habitat type where captured (plantation, more disturbed forest, and less disturbed forest), mass, and value (i.e., carcass price, as set by hunters; for carcasses not intended for sale, we asked hunters to estimate the value if they were to be sold). The main hunting methods were guns, wire snares, and pickup (collecting live animals by hand (‘pickup’), so we grouped less frequently used methods (metal trap, dog, crossbow, food poisoning, ground pit, and catapult) as ‘other’. Where hunters had already butchered an animal, we recorded the mass and value per piece. We also asked hunters about any captures consumed during the trip (we had missing data for mass in such cases). Note that we did not collect data on unsuccessful trips (i.e., no animal was captured) until December 2021. Further, all hunts within CRNP or that involved killing a protected species were illegal.

The questionnaire was administered in English via KoboToolbox (https://kf.kobotoolbox.org/). Research ethics were assessed and approved by Cambridge University’s Psychology Research Ethics Committee (applications: PRE.2020.095 and PRE.2021.071).

### Distribution of Hunting Outcomes

**Fig. 2:**
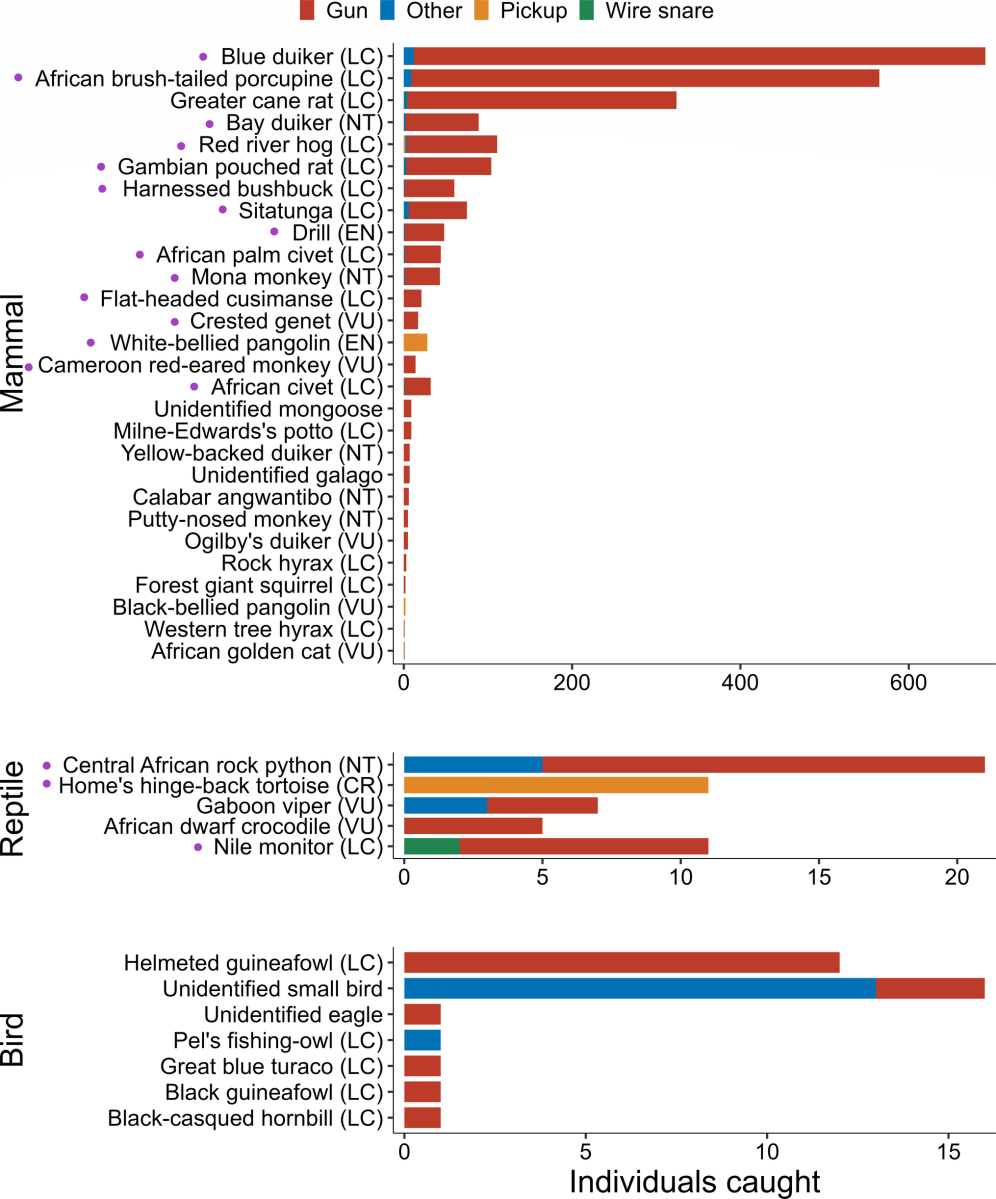
Number of individuals caught per species, plotted separately for mammals, reptiles, and birds, and colored by the associated capture methods. The figure summarizes three years (April 2020-March 2023) of wild meat offtake by 33 hunters in two communities around Nigeria’s Cross River National Park. Purple dots indicate species used in the Chi-square analysis assessing differences in proportional composition between communities. Note the different scales for each taxon. IUCN categories are in brackets: LC = Least Concern, NT = Near Threatened, VU = Vulnerable, EN = Endangered, and CR = Critically Endangered. Total offtake by mass and price are in Figures S9-10

We calculated hunting outcomes in three ways: (1) the number of individuals per species captured (hereafter count); (2) mass harvested (kg), using median mass per species, and (3) total actual or anticipated carcass price (hereafter value, in Nigerian Naira), using median value per species. We then used these measures to characterize overall patterns of offtake in a series of preliminary analyses i.e., comparing the proportional composition of offtake across species and absolute rates of offtake for each species between seasons and communities. We examined differences in the proportional composition of offtake across species (count only) between seasons (*n* = 16 species) and communities (*n* = 19 species) using Chi-square tests. We dropped species or species groups (hereafter species) with expected values < 3 and < 4 in community and season comparisons, respectively, to improve the Chi-square approximations (Fig. 2). Using Wilcoxon signed-ranks tests, we assessed differences in the absolute rates of offtake (count only) between seasons and communities. Seasonal rates were estimated by dividing count per species by the number of months per season, while community rates were calculated by dividing count per species by the number of hunter-days (using the first and last successful trip dates per hunter) multiplied by 30 to derive count per hunter-month. We then used the median hunter-month values per species (*n* = 28 species for season and community), dropping species not caught in both pair. In addition to these comparisons, we assessed the within-community spread of count, mass, and price using Gini coefficients and Lorenz curves, commonly used to assess within-population distribution of a variable (Dorfman, 1979; Lorenz, 1905). We conducted these and subsequent analyses in R version 4.2.2 (R Core Team, 2022).

### Identifying Predictors of Trip Initiation

We defined trip initiation as the decision to hunt on a given day and inferred such decisions from the start dates of each trip. Note that we restricted this analysis to January 2022-April 2023, where we recorded unsuccessful trips, thus using data from 29 hunters as others had either relocated by this time or did not provide hunter-level covariates. Computed per hunter and restricted to their first and last records, we labelled the start dates of each trip (1) and days where no trip was initiated (0) – and excluded subsequent days in multi-day trips from the analysis. We then used 1 or 0 scores for trip initiation (on *n* = 9,051 days) as the response in a binary logistic generalized linear mixed model (GLMM; Pinheiro & Bates, 2006).

With a clog-log link function to accommodate the disproportionate of the response variable (6% of 1s and 94% 0s) and a random intercept of hunter identity, we modelled trip initiation as a function of community, hunter annual household income (excluding hunting-related income; log-transformed for linearity), hunter experience (in years; log-transformed), hunter household well-being index (WBI), household size (expressed in adult male equivalents; AME), agricultural season, festivity, rainfall per trip (mm), moon phase per trip (%), and cloud cover per trip (included to account for possible attenuation of moon illumination; %). We also attempted to include another predictor: number of days since each hunter last initiated a trip; but dropped the term as our model did not converge. Agricultural season and festivity were community-level covariates, with others being hunter- or trip-level predictors (Table S1 and Fig. S1).

We derived an index of well-being by assessing each household’s access to services and affordability of goods collaboratively defined as ‘essential’ (see Detoeuf et al., 2020; Roe et al., 2023 for the protocol deployed in the study communities). We created the list of essential goods and services through workshops in four local communities in 2017 and conducted the assessment with the hunters in May 2022 using a basic necessity survey (BNS), accessible through this link https://ee.kobotoolbox.org/x/L12MXi3d. We used both income and WBI in the model because income measures financial resources, while the latter is multi-dimensional, aiming to provide a more holistic assessment of wealth, which may be purchased or gifted, such as houses, vehicles, or other valuable assets. AME measures household dietary requirements and standardizes food consumption across households using household size and composition (sex and age; Weisell & Dop, 2012). We defined agricultural season as weeks predominantly associated with farming the main crops in Cross River (cassava *Manihot esculenta*, plantain *Musa* spp., yam *Dioscorea cayenensis* spp. *rotundata*, and cocoa *Theobroma caca*) and labelled other weeks as non-agricultural season. Note that the hunters identified hunting and farming as their top two livelihood activities. We counted days as festivities if they fell within seven days of Easter, Christmas, New Year, and three traditional festivals (one in the border community and two in the distant community). All community- and hunter-level covariates were gathered in May 2022 (Table S1).

Here and in all subsequent models, we (a) examined collinearity among the predictors (before and after fitting the models) using their variance inflation factors (VIF; threshold before fitting the model = 3; Zuur et al., 2013), (b) scaled all continuous predictors, and (c) used simulated residuals (Dunn & Smyth, 1996) to visually assess model fit by plotting residuals against each predictor variable, further inspecting model fit using DHARMa package (Fig. S2; Hartig, 2022).

### Identifying Predictors of Trip Success (Number of Animals)

We assessed trip success using two measures of hunting returns: count and mass. The count model had two components, which we handled simultaneously using a truncated negative binomial GLMM (hereafter hurdle model) with linear parameterization and a random intercept of hunter identity. The first component (binary logit) predicted the probability of observing a count of at least one. The other component (conditional) explored the distribution of counts greater than zero, thus identifying predictors of the number of animals caught during successful trips. As in the previous model, we restricted the analysis to January 2022-April 2023, so again, we used data from 29 hunters (*n* = 513 trips). Further, we looked solely at gun-related offtake because we did not record the number of snares deployed and the duration of deployment, so we could not derive a measure of hunting effort (for gun-hunting this was provided by trip length). Note however that gun-hunting was responsible for 95% of all animals caught in our study.

We specified community, moon phase, cloud cover, rainfall, duration of hunting trip (in days; log-transformed), and hunter experience (log-transformed) as predictors of the probability of observing a count of at least one (binary logit) and then modelled the conditional component as a function of these variables together with AME, WBI, and income (log-transformed). Note that we removed community from the conditional component to address model singularity. Cloud cover, moon phase, and rainfall were the median values for each trip. We did not specify an offset term for duration because we assumed a non-linear accumulation of captures with increasing trip length (see Figs S3 and S4 for univariate relationships between the response and predictors variables and the model fit).

### Identifying Predictors of Trip Success (Mass of Animals)

We also explored trip success using wild meat mass harvested. Here we focused on successful trips (i.e., captures ≥ 1) and hence used data from 32 hunters from the onset of the study (April 2020-March 2023; *n* = 1,416 trips). We derived the response variable, mass per hunter per trip, by multiplying the total number of animals caught per species by their median mass before summing across species. We then assessed variation in mass caught using linear mixed effects model with a random intercept of hunter identity.

We used community, trip duration, the median moon phase, rainfall, and cloud cover values of each trip, and community, experience, income, WBI, and AME per hunter as the predictors. As above, we restricted the analysis to gun-related captures (see Figs S5 and S6 for univariate relationships between the response and predictors variables and the model fit).

### Identifying Predictors of Carcass Price

To identify predictors of carcass price, we used linear regression to model each species’ median price per kg (for *n* = 36 species) as a function of species median body mass (square root-transformed), count (log-transformed), and median palatability. We calculated price per kilogram (log-transformed) by dividing the value of whole carcasses by their corresponding mass for all species records and extracting the median value (see Fig. S7 for univariate relationships between the response and predictors). We used palatability assessments from south-east Nigeria provided through surveys of 190 hunters, 190 wild meat vendors, and 190 household members (Emogor et al., 2024b [preprint]). Given the evidence that the capture rate of species positively co-varies with the palatability of their meat (Chaves et al., 2020), we hypothesized that an increase in palatability would be associated with a rise in carcass price.

We ran two models: an additive and an interaction model. The additive model contained all covariates without interactions. The interaction model extended the additive model with an interaction between mass and count, based on an a priori hypothesis that rarely caught species (other things being equal) are sold for more money per kilogram than more commonly caught species, but the price per kilogram of more commonly caught species should generally be less sensitive to the animal’s size. We compared models using Akaike Information Criterion (AIC; Aho et al., 2014) and selected the model with the smallest AIC value.

## Results

### Characteristics of the Catch

The 33 hunters we monitored captured a total of 2,489 animals across 1,624 hunting trips conducted over 36 months (1,106 hunter-months). The total mass of their offtake was estimated at 14,700 kg with a value of ₦18,829,000 (USD 24,300 at $1 = ₦775; monthly average of ₦17,000 per hunter; Fig. 2). Gun-hunting accounted for 95% of the total count, followed by other methods (3%), pickup (i.e., by hand; 1.7%), and wire snare (0.3%). The offtake comprised 40 species (or species groups), mostly mammals (~ 96% of the total number of individuals captured), with only modest numbers of reptiles (2.7%) and birds (1.3%). Of the 36 identified species, 20 are listed as Least Concern in the International Union for Conservation of Nature’s (IUCN) Red List of Threatened Species, seven as Near Threatened, six as Vulnerable, two as Endangered, and one as Critically Endangered (Fig. 2).

Capture rate (i.e., the number of individuals caught per hunter-month) differed markedly between taxonomic classes, with mammals having the highest capture rate overall. The hunters sold 93% (13,600 kg) of harvested wild meat. The distributions of count, mass, and value among hunters in the same community were comparable between the border and distant communities, with the Gini coefficients across outcomes for each community ranging between 0.06 and 0.13: hence the distribution of hunting returns across the hunters we surveyed was relatively even (Fig. S11). In terms of the proportional composition of offtake across species (computed using Pearson’s Chi-square), we found that there was a difference in count per species between communities (χ^2^ = 180.36, df = 18, *p* < 0.001) but not between seasons (χ^2^ = 22.42, df = 14, *p* = 0.07). Of the 19 species in the community comparison, the following were caught disproportionately more than expected in the border community: African brush-tailed porcupine (*Atherurus africanus*), African palm civet (*Nandinia binotata*), bay duiker (*Cephalophus dorsalis*), blue duiker (*Philantomba monticola*), flat-headed cusimanse (*Crossarchus platycephalus*), Home’s hinged-back tortoise (*Kinixys homeana*), mona monkey (*Cercopithecus mona*), and white-bellied pangolin (*Phataginus tricuspis*). The remainder (Fig. 2) were caught disproportionately more than expected in the distant community. Further, the absolute rates of offtake between seasons revealed higher offtake rates in the wet compared to the dry season (V = 314, *n* = 28, *p* = 0.01). There was no difference in rates between communities (V = 168, *n* = 28, *p* = 0.44; Fig. S12).

### Predictors of Trip Initiation

**Fig. 3:**
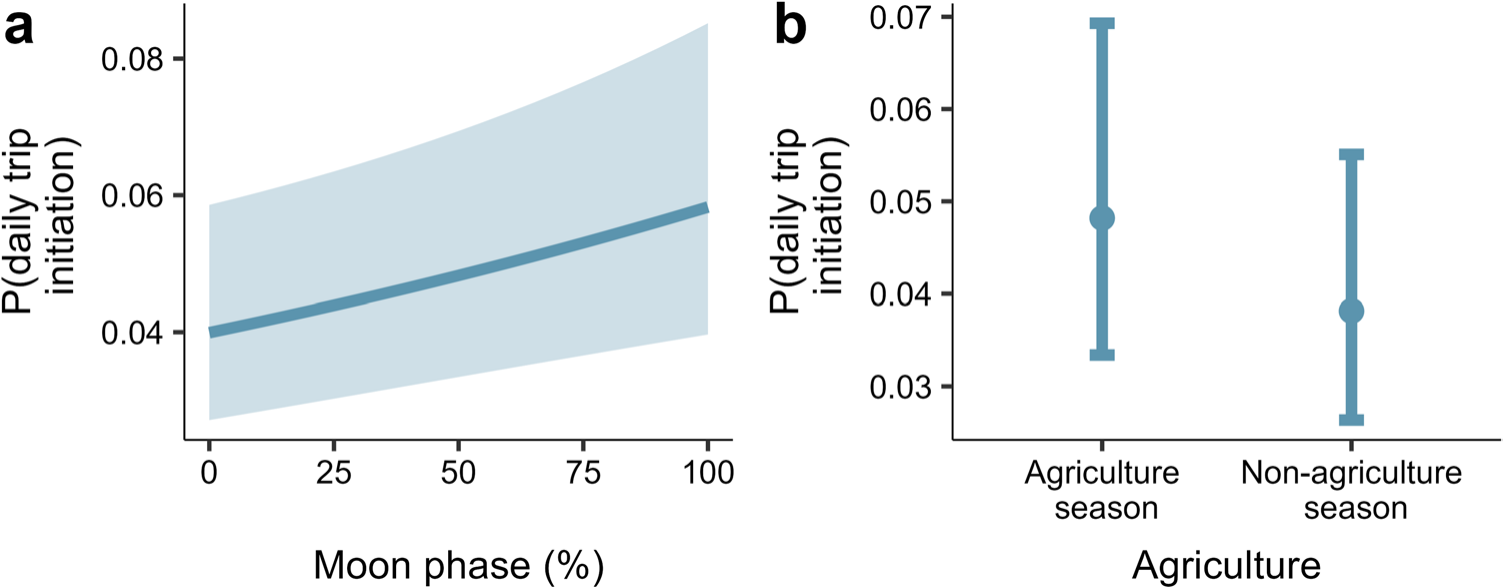
The probability of initiating a hunting trip was higher near a full moon phase (100% moon phase) than in darker periods (**a**) and during agricultural seasons (**b**). The curve is the fitted relationship, with ribbons representing 95% confidence intervals (**a**). The error bars show the effects of the variable – the circles are model predictions while the lines are whiskers are 95% confidence intervals (**b**). Data represent wild meat offtake by 29 hunters in two communities adjacent to Nigeria’s Cross River National Park (January 2022-March 2023)

The trip initiation model identified moon phase and agricultural season as predictors of whether or not a hunter embarked on a hunting trip on any given day. Moon phase was positively correlated with trip initiation, suggesting a higher probability of initiating a trip on days near a full moon (100% moon phase) than new moon (*β* = 0.14, *SE* = 0.04, *p* = 0.002; Fig. 3a). In terms of farming activity, hunters were less likely to hunt during non-agricultural seasons than in periods associated with farming (*β* = −0.24 *SE* = 0.09, *p* = 0.007; Fig. 3b, Table S2).

### Predictors of Trip Success (Number and Mass)

**Fig. 4:**
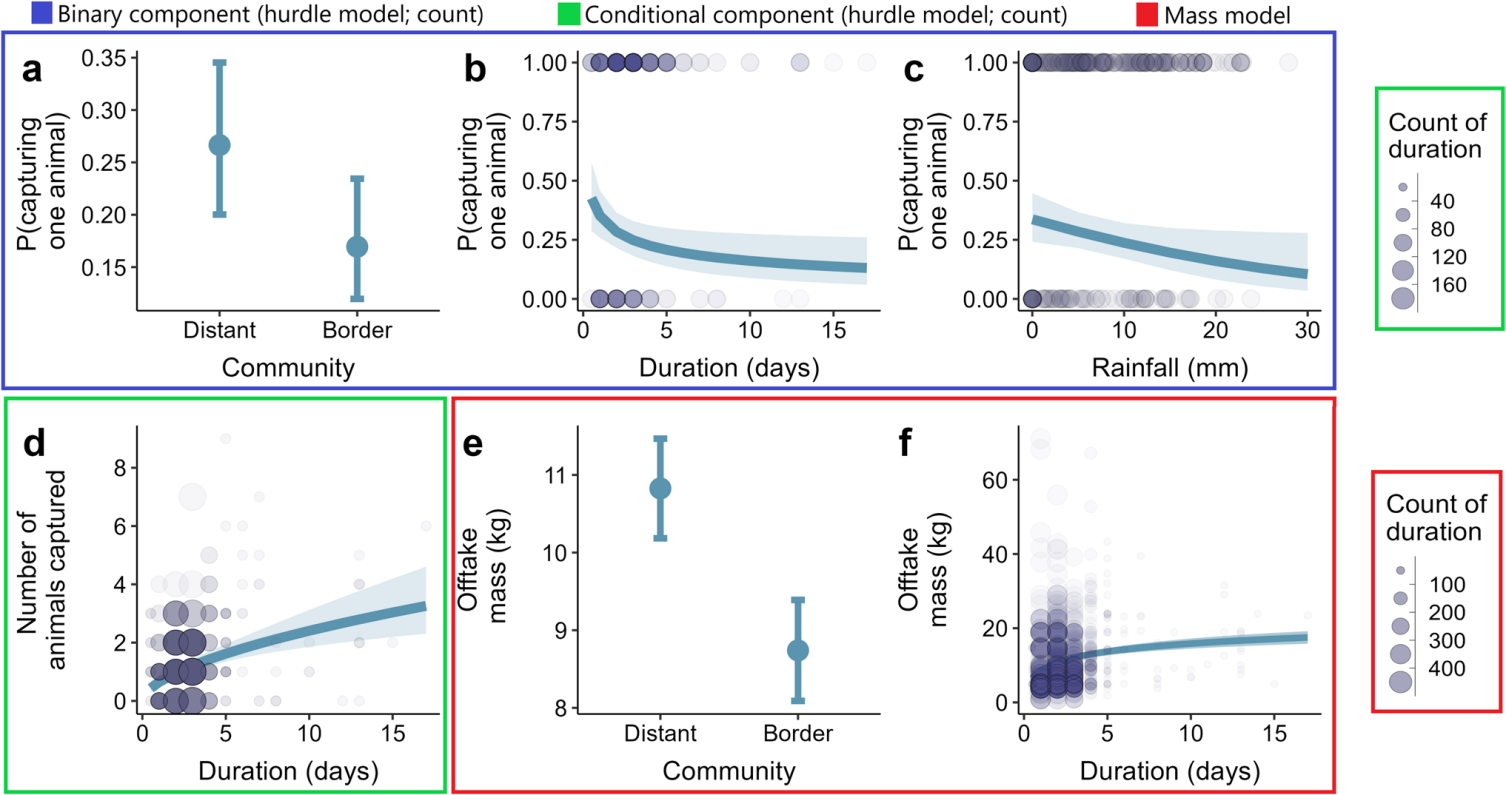
Predictors of the success of hunting trips, assessed in three ways: the probability of capturing at least one animal (binary logit component of the hurdle model; **a**-**c**), number of animals captured on trips that caught at least one (conditional component of the hurdle model; **d**), and mass harvested on successful trips (mass model; **e**-**f**) – see color codes. Error bars show predicted probability (dot) and confidence intervals (lines; **a** and b). Continuous lines are model predictions, and ribbons are 95% confidence intervals (**b**-**d**, **f**). Blue circles are raw data (circles in d and f represent count; see key) from two communities around Nigeria’s Cross River National Park, based on data from 29 hunters surveyed (January 2022-March 2023; **a**-**d**) and 32 hunters surveyed (April 2020-March 2023; **e**-**f**)

Turning to the hurdle model predicting hunting trip success, we found that hunters in the border community had a lower probability of having a successful trip (i.e., capturing at least one animal) than those in the distant community (*β* = −0.58 *SE* = 0.28, *p* = 0.04; Fig. 4a). The binary logit component of the hurdle model further identified rainfall and trip duration as predictors of the probability of having a successful trip. The model showed that the probability of capturing at least one animal was higher on days with low rainfall (*β* = −0.31, *SE* = 0.15, *p* = 0.04; Fig. 4b). Similarly, longer trips were less likely to be successful (*β* = −0.28, *SE* = 0.11, *p* = 0.01; Fig. 4c). The conditional component of the hurdle model then explored variation in the number of animals caught on those trips, which were successful. This revealed that the level of success was positively associated with trip duration (*β* = 0.40, *SE* = 0.06, *p* < 0.001; Fig. 4d), implying that, conditional on capturing one animal, hunters captured more animals the longer they hunted. No other covariate was associated with either capturing a single animal or capturing more than one after catching the first (full details in Table S3).

In the mass model of trip success, we found that hunters in the border community harvested less wild meat mass per successful trip than those in the distant community (*β* = −2.09, *SE* = 0.49, *p* < 0.001; Fig. 4e). Corroborating the findings in the conditional component of the hurdle model, the mass model also showed that longer trips were associated with higher offtake by mass (*β* = 1.82, *SE* = 0.20, *p* < 0.001; Fig. 4f). No other trip- and hunter-level covariates predicted the variation in mass harvested per trip (Table S4).

### Predictors of Carcass Price

**Fig. 5:**
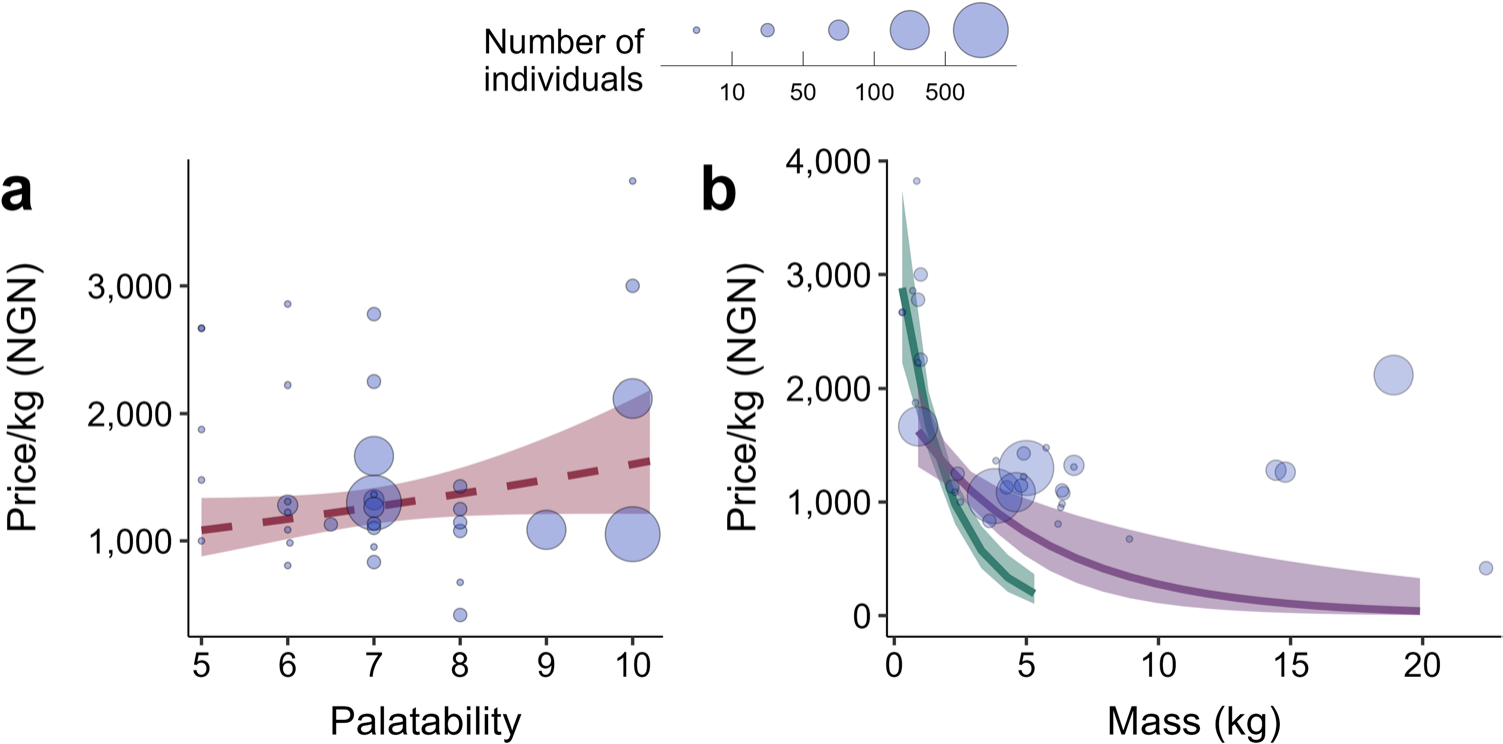
The price of species carcasses (expressed as price per kilogram; *n* = 36 species) was highest for more palatable species (**a**), with the price of rarely caught, small-bodied species higher overall and declining more sharply with mass than that of commonly caught, large-bodied species (**b**). The number of individuals captured (count) was specified as a continuous variable, with the 0.25 and 0.75 quantiles (green and purple curves, respectively) used to illustrate the interaction between count and mass (**b**). Blue circles in both panels show the observed data for each species, with the size of the circle representing count (see key). Continuous lines are model predictions, and ribbons are 95% confidence intervals. The dashed line in a represents a marginal effect (i.e., *p* = 0.08). Data are based on wild meat offtake by 33 hunters in two communities adjacent to Nigeria’s Cross River National Park (April 2020-March 2023)

The interaction model (the most parsimonious) showed evidence of a marginally significant positive association between price and palatability (*β* = 0.11, *SE* = 0.06, *p* = 0.08; Fig. 5a), suggesting that, on average, the meat of highly palatable species costs more per kg. The model also revealed a significant interaction between count and mass, whereby the price per kg was higher for rarely caught small-bodied animals and decreased as body mass increased, particularly for rarely caught species and to a lesser extent for frequently caught ones (*β* = 0.2, *SE* = 0.06, *p* < 0.001; Fig. 5b). The model explained 59% of the variance in species price (*F*_(4,31)_ = 13.42; Table S5).

### Discussion and Conclusions

To improve our understanding of the behaviors of wild meat hunters, we used hunting data collected over three years to identify predictors of daily hunting decisions and of the success of hunting trips. We also assessed predictors of carcass price, as our study hunters consumed, rather than sold, a disproportionately large fraction of their total captures. Among the hunters we followed, who were members of the community hunter association and mostly used guns to hunt, gun hunting accounted for 95% of total offtake, with mammals representing the highest offtake by count, mass, and value. The estimated value of all offtakes amounted to a monthly average of ~ ₦17,000 per hunter, representing 57% of the monthly minimum wage in Nigeria during the study (National Minimum Wage Act, 2019).

Our model of trip initiation showed a higher probability of embarking on a hunt on full moon days (Fig. 3a). This result differs from our expectation that trip initiation would be more likely during darker moon phases when animals (especially mammals) are more active at night (Prugh & Golden, 2014) and possibly more likely to be seen by hunters. The trip initiation model also revealed a higher probability of embarking on trips during periods of higher farming activity (Fig. 3b). This result differs from findings in Ghana where wild meat harvesting is more common during quiet periods for farming (Schulte-Herbrüggen et al., 2013), but matches findings in Gabon, where hunters generally use traps during period of low farming activity, switching to gun hunting during the agricultural season, as gun hunting is a more flexible activity that can be conducted after agricultural work is done (Coad, 2008). In our study landscape, hunters combine hunting and farming as their hunting grounds are interspersed with farmlands (D. Agbor, pers. obs), which means that hunting and farming may be complementary activities, with hunting from farmland requiring less travel time than initiating a trip from home.

The trip success models (count and mass) showed a relatively higher success rate in the distant community located relatively farther from CRNP (Fig. 4a, e) – a counterintuitive finding given the widespread view of protected areas as the source of wild animals (Novaro et al., 2000). Further, our first model of hunting success, exploring variation in whether trips caught any animals, showed a decrease in the probability of capturing one animal as rainfall and trip length increased (Fig. 4b-c). Paradoxically, therefore, unsuccessful trips tend to be longer than successful ones. However, among successful trips, we found that the longer the trip the more animals were caught (Fig. 4d, f). These seemingly contradictory associations between trip length and indicators of success could perhaps be explained by hunters setting a target for the minimum number of animals they expect to catch on any given trip, and then strive not to return home empty-handed. This form of loss averse decision-making is novel for wild meat hunters but has famously been observed among New York taxi drivers who work longer hours on days with relatively little work to reach some predefined daily target (Camerer et al., 1997). In the present context, it appears that hunters continue to hunt until they have caught at least one animal unless, of course, they fail to achieve even this, which would make unsuccessful trips longer. On the other hand, once a trip is successful, it appears that hunters may then be motivated to keep hunting – so that among successful trips, longer ones involve more captures.

Turning to our findings on carcass price, the positive correlation between meat palatability and carcass price suggests hunters may experience a financial incentive to target more palatable species (Fig. 5a). This result corresponds with evidence from Brazil’s Caatinga, showing that the most preferred meat species were more likely to be hunted (Chaves et al., 2020). We also found that smaller-bodied species had a higher price per kg if they were infrequently caught but that larger-bodied and more commonly caught animals fetched lower prices per kg (Fig. 5b), suggesting that small-bodied, infrequently captured species may be at a greater risk of overexploitation because they fetch higher prices. Nonetheless, this may only apply to those species captured with methods other than guns – i.e., low-cost methods, such as by hand: black-bellied pangolin (*Phataginus tetradactyla*), white-bellied pangolin, and Home’s hinge-back tortoise in this study (see ‘Pickup’ in Fig. 2) – as ‘infrequency’ in capture could be due to the avoidance of gun hunters from hunting small-bodied animals to save their ammunition for bigger game. We also found that, regardless of size, the price of whole carcasses declined as mass increased, suggesting that people are willing to pay a premium for scarce resources, especially those with limited stock (Koford & Tschoegl, 1998; Leibenstein, 1950).

The use of self-reported data in this study does raise some important caveats for our results. Our work is subject to social desirability bias, i.e., the desire to over-report socially acceptable actions (Kormos & Gifford, 2014). However, this bias may be limited in this study given that hunters volunteered data (including photographic evidence) on their involvement in illegal activities (e.g., killing federally protected species, including pangolins and drills, *Mandrillus leucophaeus*). Further, although we focused entirely on formal hunters, casual hunters account for approximately 40% of the total offtake in the landscape (Emogor et al., 2024a), so our results are potentially representative of a relatively moderate proportion of the total offtake. Finally, we are unable to draw causal results from our observational data. Rather, we suggest that the correlations we present may be useful for targeting interventions (e.g., by predicting when hunting activity may be particularly high) and identifying issues that future, more casually focused work may examine.

Our findings have several important implications for wildlife conservation and sustainable wild meat harvest promotion. First, they suggest that protected area managers might consider increasing anti-poaching patrol efforts during periods of peak agricultural activities and full moon periods. Similarly, the knowledge of a possible target-based approach to hunting could be used to design interventions to substitute this targeted outcome. Lastly, our work highlights the need to consider and monitor the impact of wild meat capture on rarely caught small-bodied species that are usually captured by hand, in addition to the medium-to-smaller-bodied duiker species typically used in assessments of offtake sustainability, given the apparent financial premium placed on rarer wild meat (Froese et al., 2022; Hongo et al., 2022; Yasuoka et al., 2015).

## Supplementary Information

Below is the link to the electronic supplementary material.

ESM 1 (DOCX 6.01 MB)

## Data Availability

The data that support the findings of this study are available on reasonable request from the corresponding author (C.A.E). The data are not publicly available as they contain information that could compromise research participant anonymity.

## References

[CR1] Abernethy, K. A., Coad, L., Taylor, G., Lee, M. E., & Maisels, F. (2013). Extent and ecological consequences of hunting in Central African rainforests in the twenty-first century. *Philosophical Transactions of the Royal Society B: Biological Sciences,**368*(1625), 20120303. 10.1098/rstb.2012.0303 10.1098/rstb.2012.0303 PMC3720024 23878333

[CR2] Aho, K., Derryberry, D., & Peterson, T. (2014). Model selection for ecologists: The worldviews of AIC and BIC. *Ecology,**95*(3), 631–636. 10.1890/13-1452.1 24804445 10.1890/13-1452.1

[CR3] Akpan, E. R., & Offem, J. (1993). *Seasonal variation in water quality of the Cross River, Nigeria*. https://www.semanticscholar.org/paper/Seasonal-variation-in-water-quality-of-the-Cross-Akpan-Offem/9ffcf63cbe74ee756ffa856903964d0b316db471

[CR4] Bello, C., Galetti, M., Pizo, M. A., Magnago, L. F. S., Rocha, M. F., Lima, R. A. F., Peres, C. A., Ovaskainen, O., & Jordano, P. (2015). Defaunation affects carbon storage in tropical forests. *Science Advances,**1*(11), e1501105. 10.1126/sciadv.1501105 26824067 10.1126/sciadv.1501105 PMC4730851

[CR5] Bowler, M., Beirne, C., Tobler, M. W., Anderson, M., DiPaola, A., Fa, J. E., Gilmore, M. P., Lemos, L. P., Mayor, P., Meier, A., Menie, G. M., Meza, D., Moreno-Gutierrez, D., Poulsen, J. R., de Souza Jesus, A., Valsecchi, J., & Bizri, E. (2020). LED flashlight technology facilitates wild meat extraction across the tropics. *Frontiers in Ecology and the Environment,**18*(9), 489–495. 10.1002/fee.2242

[CR6] Camerer, C., Babcock, L., Loewenstein, G., & Thaler, R. (1997). Labor Supply of New York City cabdrivers: One day at a Time*. *The Quarterly Journal of Economics,**112*(2), 407–441. 10.1162/003355397555244

[CR7] Chaves, L. S., Alves, R. R. N., & Albuquerque, U. P. (2020). Hunters’ preferences and perceptions as hunting predictors in a semiarid ecosystem. *Science of the Total Environment,**726*, 138494. 10.1016/j.scitotenv.2020.138494 32320877 10.1016/j.scitotenv.2020.138494

[CR8] Coad, L. M. (2008). *Bushmeat hunting in Gabon: Socio-economics and hunter behaviour* [Thesis, University of Cambridge]. https://doi.org/10/252091.

[CR9] Coad, L., Fa, J. E., Abernethy, K., Van Vliet, N., Santamaria, C., Wilkie, D., Bizri, E., Ingram, H. R., Cawthorn, D. J., & Nasi, R. (2019). *Towards a sustainable, participatory and inclusive wild meat sector*. CIFOR.

[CR10] R Core Team (2022). *R: A language and environment for statistical computing.* [Computer software]. R Foundation for Statistical Computing, Vienna, Austria. http://www.r-project.org/

[CR11] Cowlishaw, G., Mendelson, S., & Rowcliffe, J. M. (2005). Structure and operation of a Bushmeat Commodity Chain in Southwestern Ghana. *Conservation Biology,**19*(1), 139–149. 10.1111/j.1523-1739.2005.00170.x

[CR12] Davies, G. (2002). Bushmeat and International Development. *Conservation Biology,**16*(3), 587–589.

[CR13] Detoeuf, D., Wieland, M., & Wilkie, D. (2020). *Guide 2.0 to the Modified Basic Necessities Survey: Why and How to Conduct Digital-Based BNS in Conservation Landscapes*. 10.19121/2020.Report.38385

[CR14] Dorfman, R. (1979). A formula for the Gini Coefficient. *The Review of Economics and Statistics,**61*(1), 146–149. 10.2307/1924845

[CR15] Dunn, P. K., & Smyth, G. K. (1996). Randomized Quantile residuals. *Journal of Computational and Graphical Statistics,**5*(3), 236–244. 10.2307/1390802

[CR16] Emogor, C. A., Coad, L., Balmford, B., Ingram, D. J., Detoeuf, D., Fletcher Jr., R. J., Imong, I., Dunn, A., & Balmford, A. (2024a). Changes in wild meat hunting and use by rural communities during the COVID-19 socio-economic shock. *Conservation Letters, 17*(5), e13042. 10.1111/conl.13042

[CR17] Emogor, C. A., Ebri, I. B., Atsu, B. A., Ogu, D. S., Iferi, O. B., & Balmford, A. (2024b). Assessing the palatability of different meats consumed in a biodiversity hotspot using a generalisable protocol. *OSF.* Preprints. 10.31219/osf.io/q98vr

[CR18] Fa, J., Seymour, S., Dupain, J., Amin, R., Albrechtsen, L., & Macdonald, D. (2006). Getting to grips with the magnitude of exploitation: Bushmeat in the Cross-sanaga Rivers region, Nigeria and Cameroon. *Biological Conservation,**129*, 497–510. 10.1016/j.biocon.2005.11.031

[CR19] Fa, J. E., Olivero, J., Real, R., Farfán, M. A., Márquez, A. L., Vargas, J. M., Ziegler, S., Wegmann, M., Brown, D., Margetts, B., & Nasi, R. (2015). Disentangling the relative effects of bushmeat availability on human nutrition in central Africa. *Scientific Reports,*8168*5*(1). 10.1038/srep08168 10.1038/srep08168 PMC4313087 25639588

[CR20] Friant, S., Ayambem, W. A., Alobi, A. O., Ifebueme, N. M., Otukpa, O. M., Ogar, D. A., Alawa, C. B. I., Goldberg, T. L., Jacka, J. K., & Rothman, J. M. (2020). Eating Bushmeat Improves Food Security in a Biodiversity and Infectious Disease. *Hotspot EcoHealth,**17*(1), 125–138. 10.1007/s10393-020-01473-0 32020354 10.1007/s10393-020-01473-0

[CR21] Friant, S., Bonwitt, J., Ayambem, W. A., Ifebueme, N. M., Alobi, A. O., Otukpa, O. M., Bennett, A. J., Shea, C., Rothman, J. M., Goldberg, T. L., & Jacka, J. K. (2022). Zootherapy as a potential pathway for zoonotic spillover: A mixed-methods study of the use of animal products in medicinal and cultural practices in Nigeria. *One Health Outlook,**4*(1), 5. 10.1186/s42522-022-00060-3 35216623 10.1186/s42522-022-00060-3 PMC8881094

[CR22] Froese, G. Z. L., Mbélé, E., Beirne, A., Atsame, C., Bayossa, L., Bazza, C., Bidzime Nkoulou, B., Dzime, M., Ebeba, N. S., Edzidzie, J., Ekazama Koto, J., Imbomba, S., Mapio, S. M., Mabouanga, E. M., Edang, H. G. M., Metandou, E. L., Mossindji, J., Ngoboutseboue, C., Nkwele, I., & Poulsen, C. (2022). Coupling paraecology and hunter GPS self-follows to quantify village bushmeat hunting dynamics across the landscape scale. *African Journal of Ecology,**60*(2), 229–249. 10.1111/aje.12956

[CR23] Hartig, F. (2022). *DHARMa: Residual Diagnostics for Hierarchical (Multi-Level / Mixed) Regression Models.* [Computer software]. https://CRAN.R-project.org/package=DHARMa

[CR24] Hongo, S., Dzefack, Z. C. B., Vernyuy, L. N., Minami, S., Mizuno, K., Otsuka, R., Hiroshima, Y., Djiéto-Lordon, C., Nakashima, Y., & Yasuoka, H. (2022). Predicting bushmeat biomass from species composition captured by camera traps: Implications for locally based wildlife monitoring. *Journal of Applied Ecology,**59*(10), 2567–2580. 10.1111/1365-2664.14257

[CR25] Ingram, D. J., Coad, L., Milner-Gulland, E. J., Parry, L., Wilkie, D., Bakarr, M. I., Benítez-López, A., Bennett, E. L., Bodmer, R., Cowlishaw, G., Bizri, H. R. E., Eves, H. E., Fa, J. E., Golden, C. D., Iponga, D. M., Minh, N. V., Morcatty, T. Q., Mwinyihali, R., Nasi, R., & Abernethy, K. (2021). Wild Meat Is Still on the Menu: Progress in Wild Meat Research, Policy, and Practice from 2002 to 2020. *Annual Review of Environment and Resources*, *46*(1), null. 10.1146/annurev-environ-041020-063132

[CR26] Koford, K., & Tschoegl, A. E. (1998). The market value of rarity. *Journal of Economic Behavior & Organization,**34*(3), 445–457. 10.1016/S0167-2681(97)00084-X

[CR27] Kormos, C., & Gifford, R. (2014). The validity of self-report measures of proenvironmental behavior: A meta-analytic review. *Journal of Environmental Psychology,**40*, 359–371. 10.1016/j.jenvp.2014.09.003

[CR28] L’Roe, J., Detoeuf, D., Wieland, M., Ikati, B., Enduyi Kimuha, M., Sandrin, F., Sukari, A., Nzale Nkumu, O., Kretser, J., & Wilkie, D. (2023). Large-scale monitoring in the DRC’s Ituri forest with a locally informed multidimensional well-being index. *World Development,**169*, 106284. 10.1016/j.worlddev.2023.106284

[CR29] Leibenstein, H. (1950). Bandwagon, Snob, and Veblen effects in the theory of consumers’ demand. *The Quarterly Journal of Economics,**64*(2), 183–207. 10.2307/1882692

[CR30] Lescuyer, G., & Nasi, R. (2016). Financial and economic values of bushmeat in rural and urban livelihoods in Cameroon: Inputs to the development of public policy. *The International Forestry Review,**18*, 93–107.

[CR31] Lorenz, M. O. (1905). Methods of measuring the concentration of Wealth. *Publications of the American Statistical Association,**9*(70), 209–219. 10.1080/15225437.1905.10503443

[CR32] Myers, N., Mittermeier, R. A., Mittermeier, C. G., da Fonseca, G. A. B., & Kent, J. (2000). Biodiversity hotspots for conservation priorities. *Nature,**403*(6772). 10.1038/35002501. Article 6772. 10.1038/35002501 10706275

[CR33] National Minimum Wage Act (2019). http://www.ilo.org/dyn/natlex/natlex4.detail?p_lang=&p_isn=111617&p_classification=12.02

[CR34] Novaro, A. J., Redford, K. H., & Bodmer, R. E. (2000). Effect of Hunting in Source-Sink Systems in the Neotropics. *Conservation Biology,**14*(3), 713–721.

[CR35] Pinheiro, J., & Bates, D. (2006). *Mixed-Effects Models in S and S-PLUS*. Springer Science & Business Media.

[CR36] Prugh, L. R., & Golden, C. D. (2014). Does moonlight increase predation risk? Meta-analysis reveals divergent responses of nocturnal mammals to lunar cycles. *Journal of Animal Ecology,**83*(2), 504–514. 10.1111/1365-2656.12148 24102189 10.1111/1365-2656.12148

[CR37] Riddell, M., Maisels, F., Lawrence, A., Stokes, E., Schulte-Herbrüggen, B., & Ingram, D. J. (2022). Combining offtake and participatory data to assess the sustainability of a hunting system in northern Congo. *African Journal of Ecology,**60*(2), 250–267. 10.1111/aje.13001

[CR38] Schulte-Herbrüggen, B., Cowlishaw, G., Homewood, K., & Rowcliffe, J. M. (2013). The importance of bushmeat in the livelihoods of west African cash-crop farmers living in a faunally-depleted landscape. *PloS One,**8*(8), e72807. 10.1371/journal.pone.0072807 23977355 10.1371/journal.pone.0072807 PMC3745405

[CR39] Sirén, A. H., & Wilkie, D. S. (2016). The effects of ammunition price on subsistence hunting in an amazonian village. *Oryx,**50*(1), 47–55. 10.1017/S003060531400026X

[CR40] Torres, P. C., Morsello, C., Orellana, J. D. Y., Almeida, O., de Moraes, A., Chacón-Montalván, E. A., Pinto, M. A. T., Fink, M. G. S., Freire, M. P., & Parry, L. (2022). Wildmeat consumption and child health in Amazonia. *Scientific Reports,**12*(1). 10.1038/s41598-022-09260-3 PMC8986765 35388037

[CR41] Weisell, R., & Dop, M. C. (2012). The Adult Male Equivalent Concept and its application to Household Consumption and expenditures surveys (HCES). *Food and Nutrition Bulletin,**33*(3_suppl2), S157–S162. 10.1177/15648265120333S203 23193766 10.1177/15648265120333S203

[CR42] Yasuoka, H., Hirai, M., Kamgaing, T., Dzefack, Z., Kamdoum, E., & Bobo, K. (2015). Changes in the composition of hunting catches in southeastern Cameroon: A promising approach for collaborative wildlife management between ecologists and local hunters. *Ecology and Society,**20*(4). 10.5751/ES-08041-200425

[CR43] Zuur, A. F., Hilbe, J. M., & Ieno, E. N. (2013). *A Beginner’s Guide to GLM and GLMM with R*. Highland Statistics Ltd.

