# Supplementary material for: Predictors of Frequency and Success of Wild Meat Hunting Trips and Carcass Prices in an African Biodiversity Hotspot
Source: Hum Ecol Interdiscip J. 2025 Jan 25;53(1):41–51. doi: 10.1007/s10745-025-00572-2 (PMC11971138; doi:10.1007/s10745-025-00572-2)
Supplement: Supplementary file 1 — (DOCX 6.01 MB) [file 10745_2025_572_MOESM1_ESM.docx]

Predictors of Frequency and Success of Wild Meat Hunting Trips and Carcass Prices in an African Biodiversity Hotspot

Charles A. Emogor, Daniel J. Ingram, Andrew Balmford, Robert J. Fletcher, Jr, Diane Detoeuf, Ben Balmford, Dan O. Agbor, and Lauren Coad.

Charles A. Emogor ([emogorcharles@gmail.com](mailto:emogorcharles@gmail.com))

# **Appendix A: Tables**

**Table S1:** Description of the variables used to predict frequency and success of wild meat hunting.

| Variable description | Type | Model | Assumption and/or justification | Source(s) |
| --- | --- | --- | --- | --- |
| Adult male equivalent (AME): Measure of household dietary requirement (Weisell & Dop, 2012). It standardizes food consumption using household size (number) and composition (sex and age). | Continuous | Initiation and success models^†^ | Households with higher AME require more daily protein (Godoy et al., 2010), such that hunters in these households will hunt more frequently. Note that the hunters and their households consume wild meat. | Study data (supplementary survey) |
| Agricultural season (two levels: Agriculture or Non-agriculture). | Categorical | Initiation model | In a landscape characterized by small-scale farming, agricultural demands will affect hunter’s decision to hunt such that hunters will embark on less trips during periods of peak farming activities. | Study data (supplementary survey) |
| Cloud cover: Median night cloud cover between the end and start dates of each hunting trip (%) derived for ~20 km^2^ encompassing the study communities and surrounding forests (hunting grounds). Daily records at ~3 km spatial resolution. | Continuous | Initiation and success models | Clouds attenuate moon brightness (Krieg, 2021), hence the effect of moonlight on hunter behavior will vary depending on the cloud cover. | CM SAF; Satellite Application Facility on Climate Monitoring (Meirink, Jan Fokke et al., 2022). |
| Duration: Difference between the end and start dates of each hunting trip per hunter (in days). | Continuous | Success model | Proxy of hunting effort. Longer trips are correlated with an higher capture (Jones et al., 2020). | Study data |
| Experience: The number of years hunters have been involved in gun-hunting. | Continuous | Success model | Hunting skill changes with hunter age (Koster et al., 2019). Using experience as a proxy of skill, the probability of trip success and the number of animals captured will be predicted by the experience of the hunter. | Study data (supplementary survey). |
| Festivity (two levels: Festive and Non-festive) | Categorical | Initiation model | There is a likelihood of increased hunting incidents immediately before festivals for meat and income and after festivals to compensate for resources spent during festive periods. | Study data (supplementary survey). |
| Hunter | Categorical (random intercept) | Initiation and success models | To account for dependency in the data. | Study data |
| Income: Annual household non-hunting related income (in Naira). We a) asked hunters to estimate monthly income from hunting, agriculture, timber trade, business, employment, and trade of non-timber forest products for the last 12 months; b) multiplied each income source by the number of associated months, and; extracted income-related hunts before summing to obtain annual income. | Continuous | Initiation and success models^†^ | Since hunting in the landscape is livelihood option, hunters in households with comparatively low income will hunt more often. | Study data (supplementary survey). |
| Moon phase: Median moon phase between the end and start dates of each hunting trip (%). Daily records at ~1 km spatial resolution. | Continuous | Initiation and success models | Moon phase can predict hunting success by attenuating the brightness of animals’ eyeshine, reducing detection by hunters. Moonlight also suppresses activities of nocturnal animals (Prugh & Golden, 2014). | NASA; National Aeronautics and Space Administration (SVS, 2021). |
| Rainfall: Median rainfall between the end and start dates of each hunting trip (mm) for the same spatial extent used to derive cloud cover data. Daily records at ~4 km spatial resolution. |  | Initiation and success models | Ungulate abundance at different life stages co-varies with rainfall (Ogutu et al., 2008). High rain may also interfere with gun-hunting but could underpin the decision to hunt as agricultural activities are mostly conducted during low rainfall periods. | TAMSAT; Tropical Applications of Meteorology using Satellite data and ground-based observations (Maidment et al., 2014, 2017; Tarnavsky et al., 2014). |
| Well-being index (WBI; proportion): Measure of household socio-economic security (i.e., wealth) expressed as access and affordability of collaboratively defined basic necessities (Detoeuf et al., 2020). |  | Initiation and success models^†^ | Household wealth predicts wild meat consumption (Brashares et al., 2011; Godoy et al., 2010). | Study data (supplementary survey). |

^†^ Conditional part of the hurdle model only.

**Table S2:** Binary logistic model of daily trip initiation. Number of observations = 9,051 days across 15 months (January 2022-March 2023; number of hunters = 29).

| Random effect |  |  |  |  |
| --- | --- | --- | --- | --- |
| Groups | Term | Variance | Std. dev |  |
| Hunter ID | Intercept | 0.18 | 0.42 |  |
|  |  |  |  |  |
| Fixed effects |  |  |  |  |
| Terms | Estimate (β) | Std. error (*SE*) | z value | *P* value |
| (Intercept) | -3.01 | 0.19 | -15.75 | < 0.001 |
| Community: Border | 0.19 | 0.19 | 0.98 | 0.33 |
| Moon phase | 0.14 | 0.04 | 3.13 | 0.002 |
| Cloud cover | 0.05 | 0.05 | 1.03 | 0.31 |
| Rainfall | -0.05 | 0.05 | -1.10 | 0.27 |
| AME | 0.10 | 0.10 | 0.97 | 0.33 |
| WBI | -0.08 | 0.10 | -0.88 | 0.38 |
| Agriculture: Non-agric season | -0.24 | 0.09 | -2.71 | 0.007 |
| Festivity: Non-festive | 0.11 | 0.14 | 0.80 | 0.42 |
| Hunting experience (log | -0.03 | 0.10 | -0.33 | 0.74 |
| Income (log) | -0.13 | 0.10 | -1.34 | 0.18 |

**Table S3:** Hurdle model predicting the success of hunting trips. The logit binary component modelled predictors of hunting trip success (i.e., whether at least one animal was captured), while the conditional component examined predictors of the level of success withing successful trips (i.e., the number of animals caught having caught one). Number of observations = 513 trips across 15 months (January 2022-March 2023; number of hunters = 29).

| **Logit binary**  Random effect |  |  |  |  |
| --- | --- | --- | --- | --- |
| Groups | Name | Variance | Std. Dev. |  |
| Hunter ID | Intercept | 0.16 | 0.40 |  |
|  |  |  |  |  |
| Fixed effects |  |  |  |  |
| Terms | Estimate (*β*) | Std. Error (*SE*) | z value | *P* value |
| Intercept | -1.01 | 0.19 | -5.32 | < 0.001 |
| Community: Border | -0.58 | 0.28 | -2.06 | 0.04 |
| Moon phase | -0.15 | 0.11 | -1.35 | 0.18 |
| Cloud cover | 0.15 | 0.14 | 1.05 | 0.29 |
| Rainfall | -0.31 | 0.15 | -2.03 | 0.04 |
| Duration (log) | -0.28 | 0.11 | -2.48 | 0.01 |
| Hunting experience (log) | 0.17 | 0.13 | 1.28 | 0.20 |
|  |  |  |  |  |
| **Conditional**  Random effect |  |  |  |  |
| Groups | Name | Variance | Std. Dev. |  |
| Hunter ID | Intercept | 0.03 | 0.18 |  |
|  |  |  |  |  |
| Fixed effects |  |  |  |  |
| Terms | Estimate (β) | Std. Error (*SE*) | z value | *P* value |
| Intercept | 0.07 | 0.10 | 0.68 | 0.50 |
| Moon phase | 0.00 | 0.06 | -0.03 | 0.98 |
| Cloud cover | -0.08 | 0.08 | -0.99 | 0.32 |
| Rainfall | -0.03 | 0.08 | -0.40 | 0.69 |
| Duration (log) | 0.35 | 0.06 | 5.58 | < 0.001 |
| WBI | 0.07 | 0.08 | 0.94 | 0.35 |
| Hunting experience (log) | -0.12 | 0.07 | -1.65 | 0.10 |
| AME | 0.01 | 0.07 | 0.07 | 0.95 |
| Non-hunt income (log) | -0.07 | 0.07 | -0.94 | 0.35 |

**Table S4**: Linear mixed effects model explaining the mass of wild meat harvested per trip. Number of observations = 1416 (number of hunters = 32).

| Random effects |  |  |  |  |  |
| --- | --- | --- | --- | --- | --- |
| Groups | Name | Variance | Std. Dev |  |  |
| Hunter ID | Intercept | 0.27 | 0.52 |  |  |
| Residual |  | 54.13 | 7.36 |  |  |
|  |  |  |  |  |  |
| Fixed effects |  |  |  |  |  |
| Terms | Estimate (*β*) | Std. Error (*SE*) | Degree of freedom | *T* value | *P* value |
| Intercept | 10.83 | 0.33 | 16.19 | 33.09 | < 0.001 |
| Community: Border | -2.09 | 0.49 | 23.63 | -4.27 | < 0.001 |
| Moon phase | -0.26 | 0.20 | 1404.06 | -1.31 | 0.19 |
| Cloud cover | -0.46 | 0.24 | 1403.54 | -1.93 | 0.05 |
| Rainfall | -0.25 | 0.24 | 1405.90 | -1.04 | 0.30 |
| WBI | -0.06 | 0.24 | 26.31 | -0.24 | 0.81 |
| AME | 0.21 | 0.23 | 13.83 | 0.91 | 0.38 |
| Duration (log) | 1.82 | 0.20 | 1405.17 | 8.91 | < 0.001 |
| Hunting experience (log) | -0.07 | 0.24 | 15.44 | -0.30 | 0.77 |
| Income (log) | -0.11 | 0.24 | 17.26 | -0.47 | 0.64 |

**Table S5**: Linear model predicting variation in carcass price (n = 36 species).

| Term | Estimate (*β*) | Std. error (*se*) | *T* value | *P* value |
| --- | --- | --- | --- | --- |
| Intercept | 7.15 | 0.06 | 131.12 | < 0.001 |
| Count | -0.0003 | 0.07 | -0.005 | 0.10 |
| Mass | -0.36 | 0.06 | -6.36 | < 0.001 |
| Palatability | 0.11 | 0.06 | 1.84 | 0.08 |
| Count: mass | 0.24 | 0.06 | 4.07 | < 0.001 |

# **Appendix B: Equations**

Equation for the model predicting trip initiation is given by

${Trip initiation}_{ijkl}= \beta_{0}+\beta_{1}{AME}_{i}+\beta_{2}{WBI}_{i}+ \beta_{3}{log(Experience}_{i}) +\beta_{4}{log(Income}_{i})+\beta_{5}{Community}_{ij}+ +\beta_{6}{Agriculture}_{ijk}+ \beta_{7}{Festivity}_{ijk}+ \beta_{8}{Moon phase}_{ijkl}+\beta_{9}{Cloud cover}_{ijkl}+\beta_{10}{Rainfall}_{ijkl}+ɑ_{ijkl} (1)$

where ${Trip initiation}_{ijkl}$ describes whether or not hunter *i* from community *j* in landscape *k* initiated a trip on day *l*; $\beta_{0}$ is the intercept; $\beta_{1-10}$ are the slopes of the respective predictors; and $ɑ_{ijkl}$ is a random intercept; and we assume that the errors follow a binomial distribution. We fitted the model using lme4 package (Bates et al., 2015).

The equation for the binary component of the hurdle model (trip success by number of animals captured) is given by

${Number captured}_{ijk}= \beta_{0}+ \beta_{1}{log\left( Experience \right)}_{i} +\beta_{2}{log \left( Income \right)}_{i}+ \beta_{3}{Community}_{ij}+ \beta_{4}{Moon phase}_{ijk}+\beta_{5}{Cloud cover}_{ijk}+\beta_{6}{Rainfall}_{ijk}+ɑ_{ijk} (2)$

where ${Number captured}_{ijk}$ is whether at least one animal was captured by hunter *i* from community *j* on trip *k*; $\beta_{0}$ is the intercept; $\beta_{1-6}$ are the slopes of the respective predictors; $ɑ_{ijk}$ is random intercept; we assume that the errors follow a binomial distribution.

The equation for the conditional component of the hurdle model is given by

${Number captured}_{ij}= \beta_{0}+\beta_{1}{AME}_{i}+\beta_{2}{WBI}_{i}+ \beta_{3}{log\left( Experience \right)}_{i} +\beta_{4}{log\left( Income \right)}_{i}+\beta_{5}{log\left( Duration \right)}_{ij}+\beta_{6}{Moon phase}_{ij}+\beta_{7}{Cloud cover}_{ij}+\beta_{8}{Rainfall}_{ij}+ɑ_{ij} (3)$

where ${Number captured}_{ij}$ (≥ 1) is the number of animals across species captured by hunter *i* on successful trips *j*; $\beta_{0}$ is the intercept; $\beta_{1-8}$ are the slopes of the respective predictors; $ɑ_{ij}$ is random intercept; and we assumed that the errors follow a negative binomial distribution. We fitted the hurdle model using glmmTMB package (Brooks et al., 2017).

The equation for the mass model is given by

${Mass harvested}_{ijk}= \beta_{0}+\beta_{1}{AME}_{i}+\beta_{2}{WBI}_{i}+ \beta_{3}{log \left( Experience \right)}_{i} +\beta_{4}{log\left( Income \right)}_{i}+\beta_{5}{Community}_{ij}+ \beta_{6}{log\left( Duration \right)}_{ijk}+\beta_{7}{Moon phase}_{ijk}+\beta_{8}{Cloud cover}_{ijk}+\beta_{9}{Rainfall}_{ijk}+ɑ_{ijk} (4)$

where ${Mass harvested}_{ij}$ (> 0) is the mass of animals harvested by hunter *i* from community *j* on trip *k*; $\beta_{0}$ is the intercept; $\beta_{1-9}$ are the slopes of the respective predictors; $ɑ_{ijk}$ is random intercept; and we assumed that the errors follow Gaussian distribution. We fitted the hurdle model using lme4 package.

The equation for the interaction model predicting carcass price is given by

$${log(Carcass price)}_{i}=\beta_{0}+\beta_{1}{Palatability}_{i}+{log(\beta}_{2}{Count}_{i})*{sqr(\beta}_{3}{Mass}_{i}) (5)$$

where ${Carcass price}_{i}$ is the median price of the whole carcass of *i* species; $\beta_{0}$ is the intercept; $\beta_{1-3}$ are the slopes of the respective predictors; and we assume that the errors follow a normal distribution. We fitted the model and assessed model fit using the stats package.

# **Appendix C: Figures**


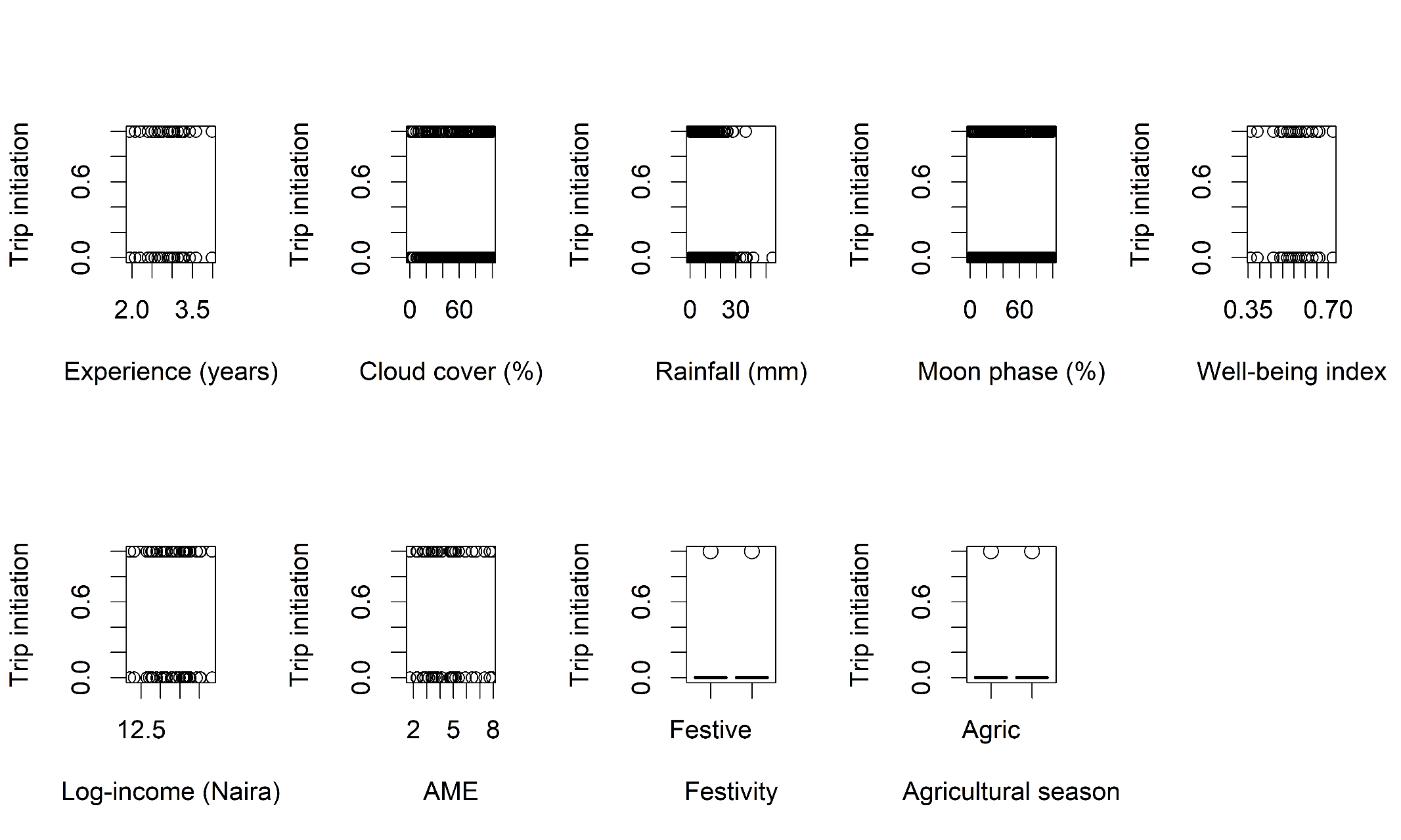


**Figure S1:** The distribution of trip initiation variables (only festivity and agricultural season are categorical – horizontal dark lines are median values). The circles are raw data of trip initiation on any given day (1 for initiation and 0 for non-initiation).


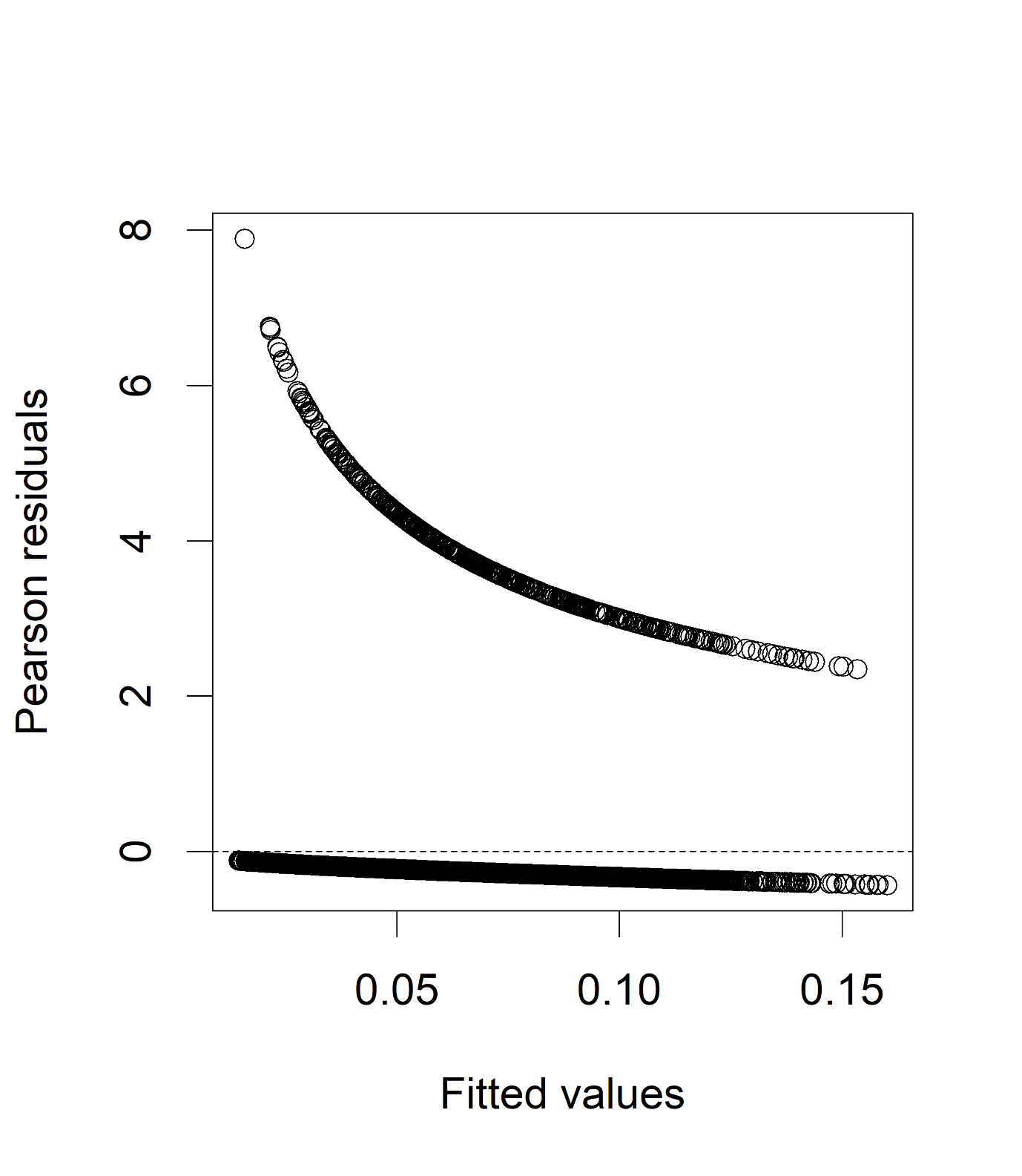


**Figure S2:** Residual of the model predicting trip initiation. The reasonably similar distribution of the Pearson residuals around 0, shown by the dotted horizontal line, suggests a good model fit.


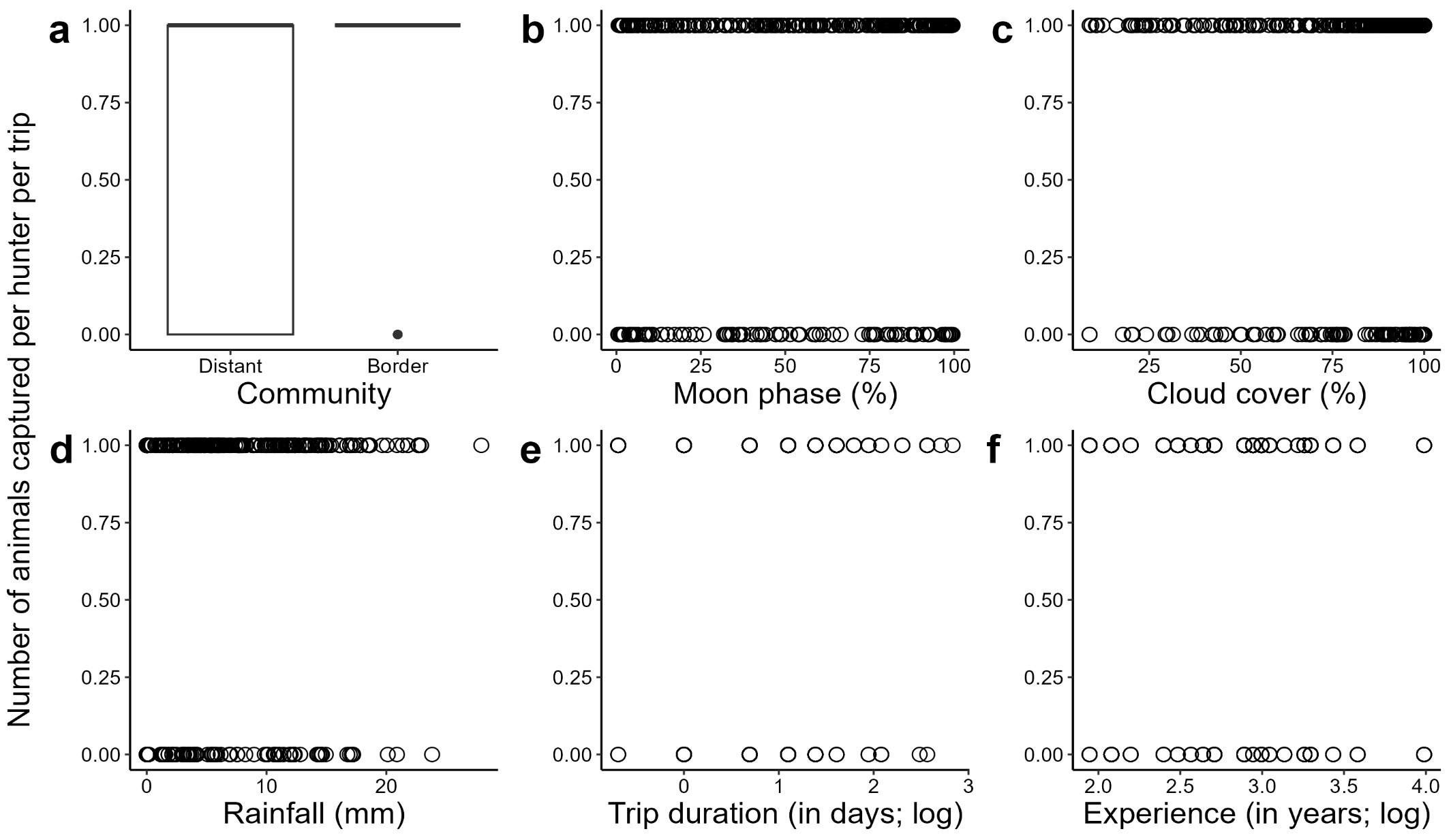
**Figure S3a:** The distribution of the predictors used in the binary component of the hurdle model of trip success. Predictor names are given in the y-axis labels. (a) shows a boxplot, with the thick horizontal line showing the median value and the rectangle representing the interquartile range (IQR). Individual dots indicate outliers (a). Circles are raw data points (b-f).


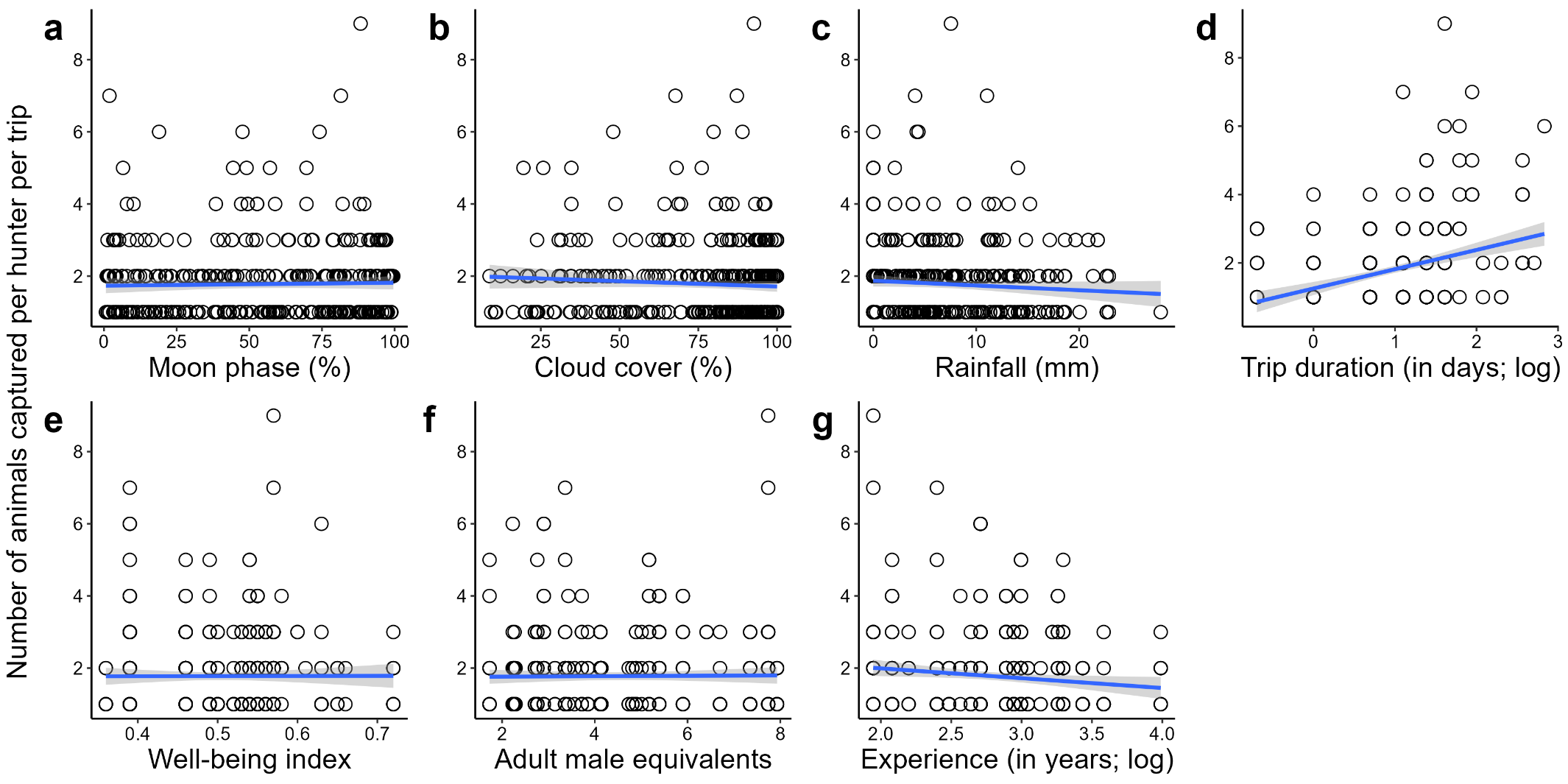


**Figure S3b**: The distribution of the predictors used in the conditional component (i.e., count above zero) of the hurdle model examining trip success. Predictor names are given in the y-axis labels. Blue lines represent the corresponding relationships (using the linear model smoother function in R) and grey ribbon are 95% credible interval. Circles are raw data points .


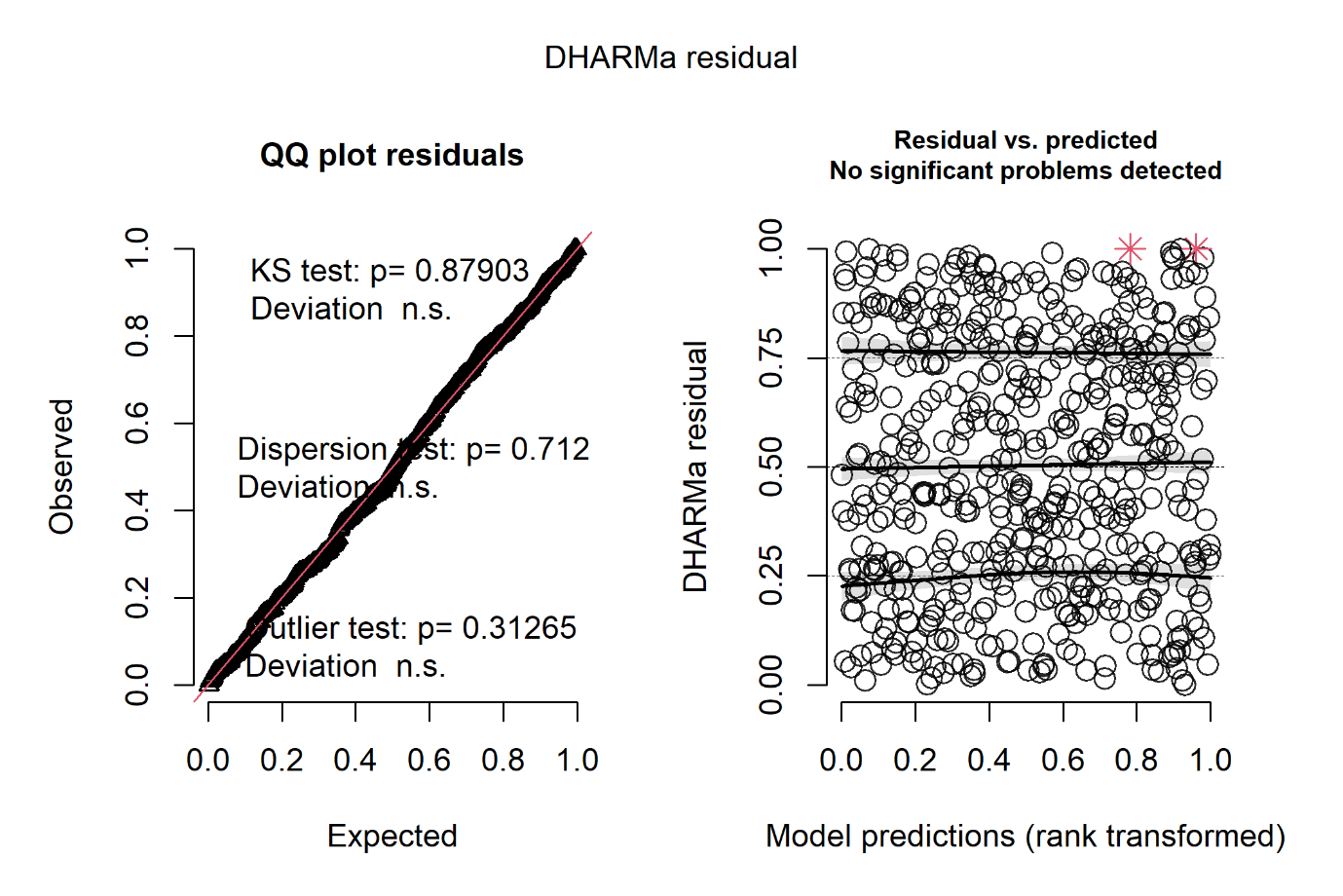


**Figure S4**: Residual plot of the hurdle model of trip success trip. The left panel shows the quantiles of the residuals (y axis) against the quantiles in the data. The plot suggests normality of the residuals as the points (black diagonal line) follow the red line. Deviations, dispersion, and outlier tests were non-significant. The right panel shows the scaled quantile residuals versus fitted values combined for all predictors. The red asterisks are outliers. The plot was made using DHARMa R package (Hartig, 2022).


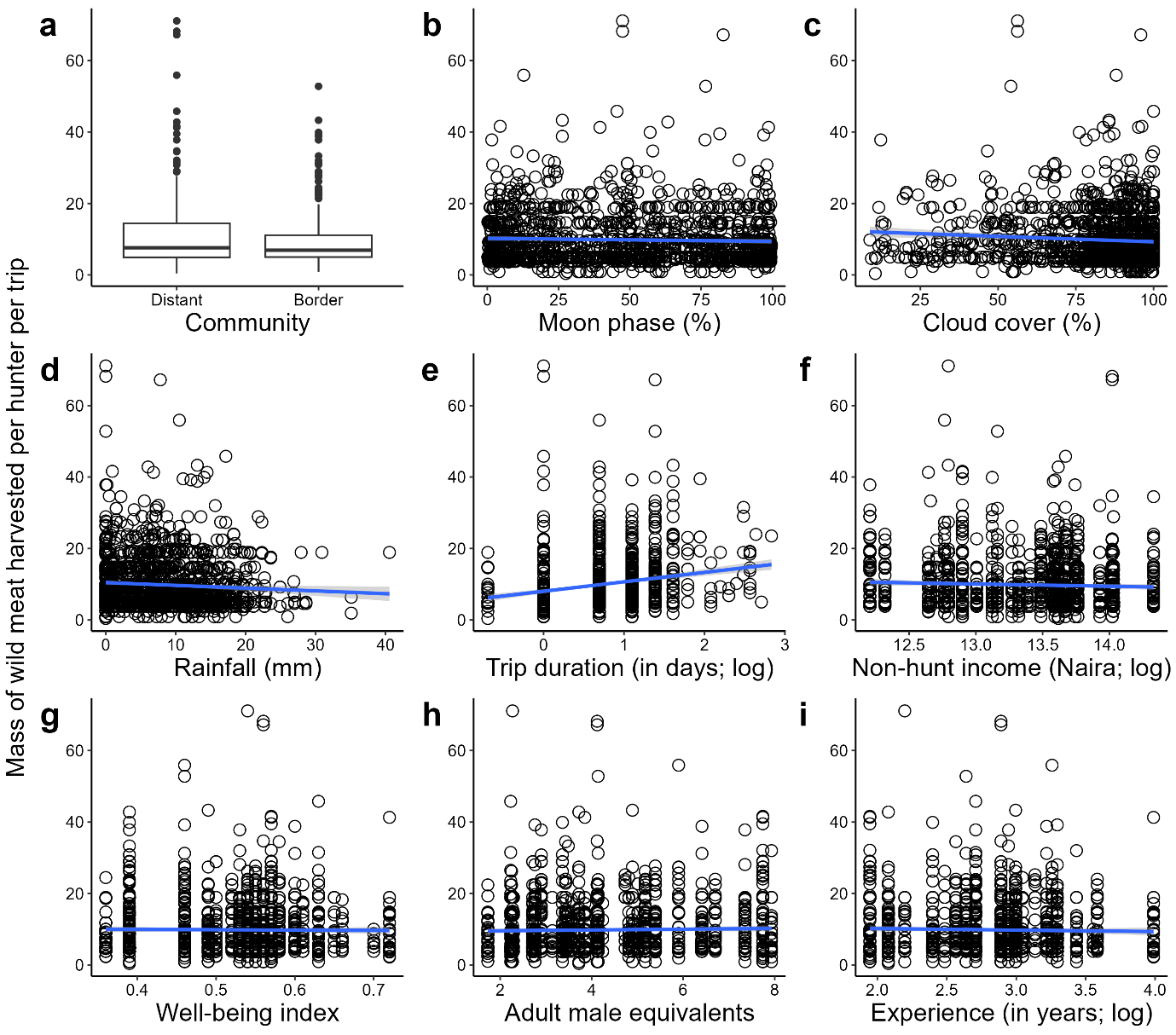


**Figure S5**: The distribution of the predictors in the model explaining the mass of wild meat harvested per trip. Predictor names are given in the y-axis labels. In a, each boxplot corresponds to the average offtake of a species from monitored hunters in a community, with the rectangle box representing the interquartile range (IQR) and the vertical line showing the distribution’s median. The lines (whiskers) extending from the boxplot show 1.5 times the IQR from the box and individual dots beyond the whiskers indicate outliers (a). Blue lines represent the corresponding relationships (using the linear model smoother function in R) and grey ribbon are 95% credible interval (b-i). Circles are raw data points.


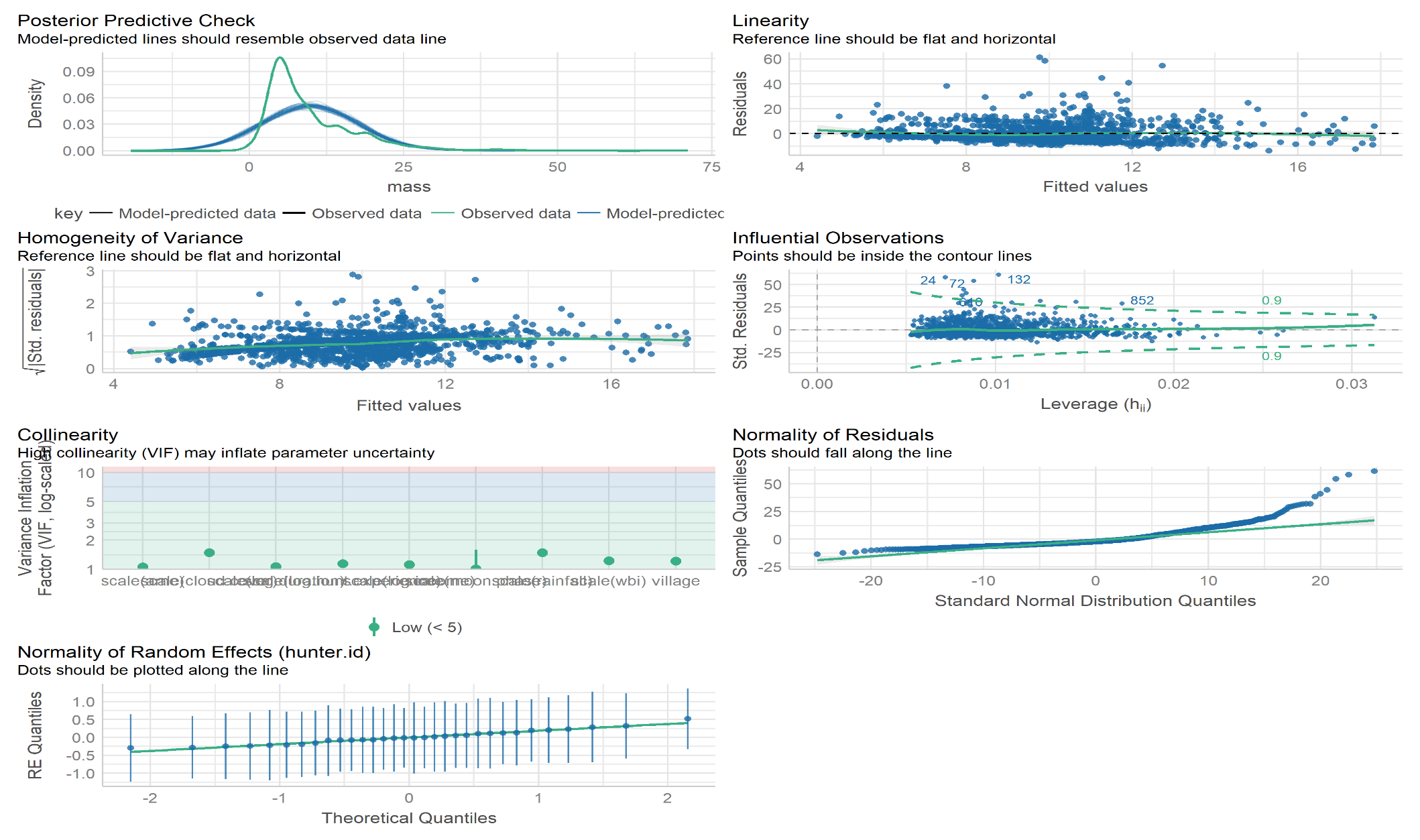


**Figure S6:** Diagnostics of the model explaining the mass of wild meat harvested per trip. Diagnostic parameters and interpretation of the plot are provided on top of each panel. Model assessment conducted using Performance package .


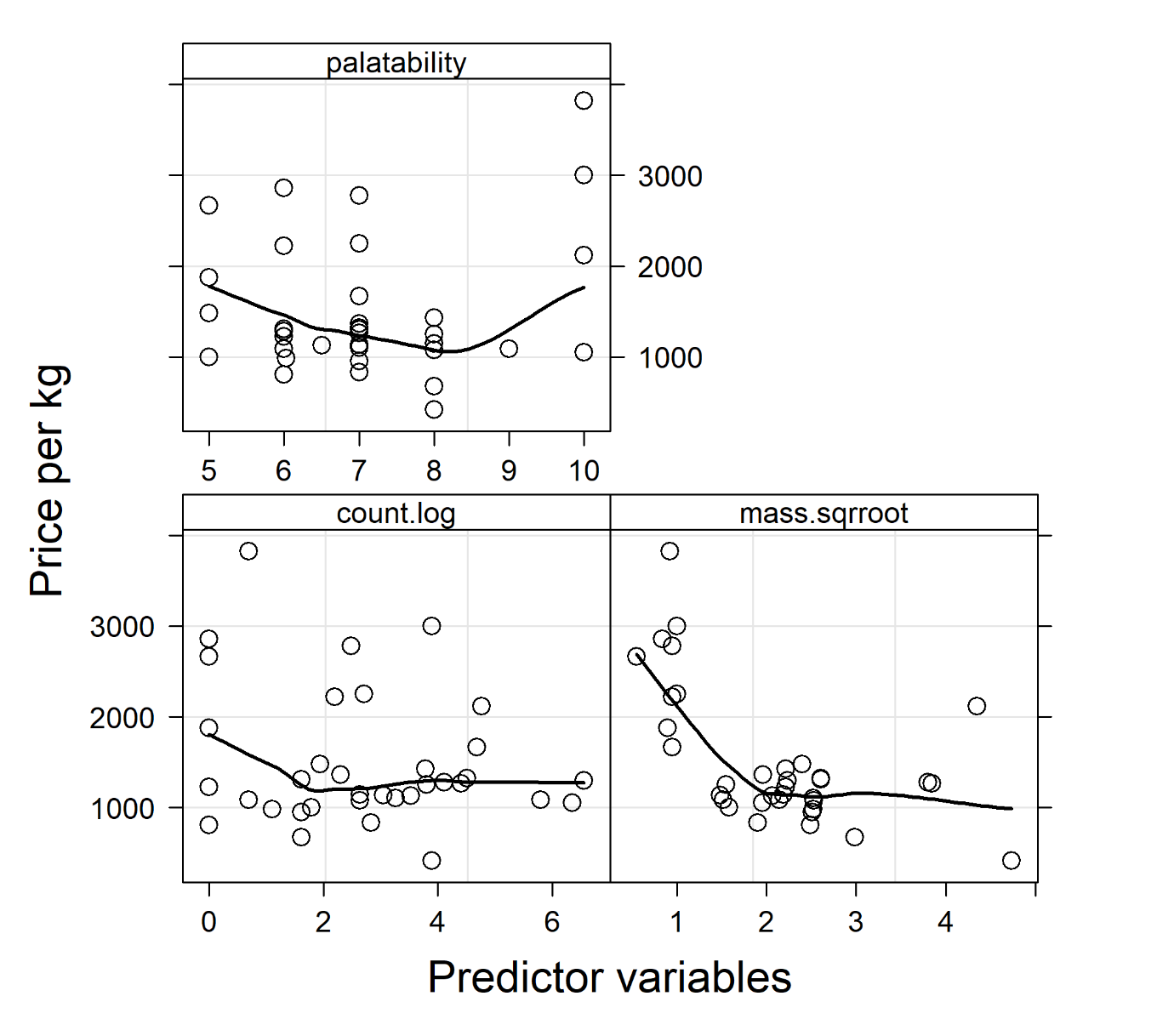


**Figure S7**: Relationships between price per kilogram and predictors used to examine correlates of species price. The panels are labelled according to the corresponding covariate. Note that count was log-transformed while mass was square root-transformed. The y-axis values apply to all plots, while the x-axis values change to reflect the range of the corresponding covariate. A LOWESS (locally weighted scatterplot smoothing) curve was added to aid visual interpretation. The plot was made with the *Myxyplot* function adapted from Zuur et al. (2013).


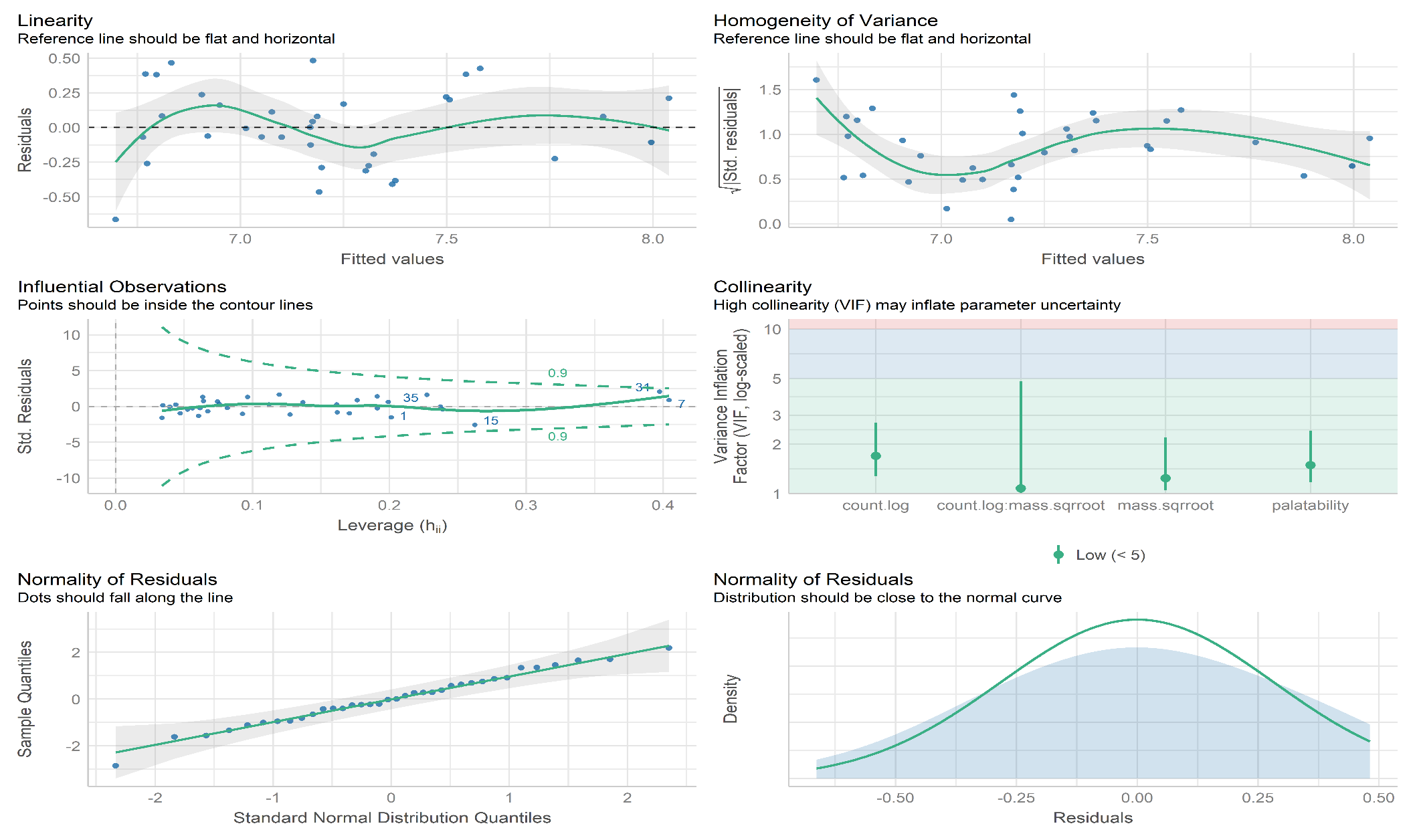


**Figure S8:** Diagnostics of the model predicting carcass price. Diagnostic parameters and interpretation of the plot are provided on top of each panel. Model assessment was conducted using Performance package.


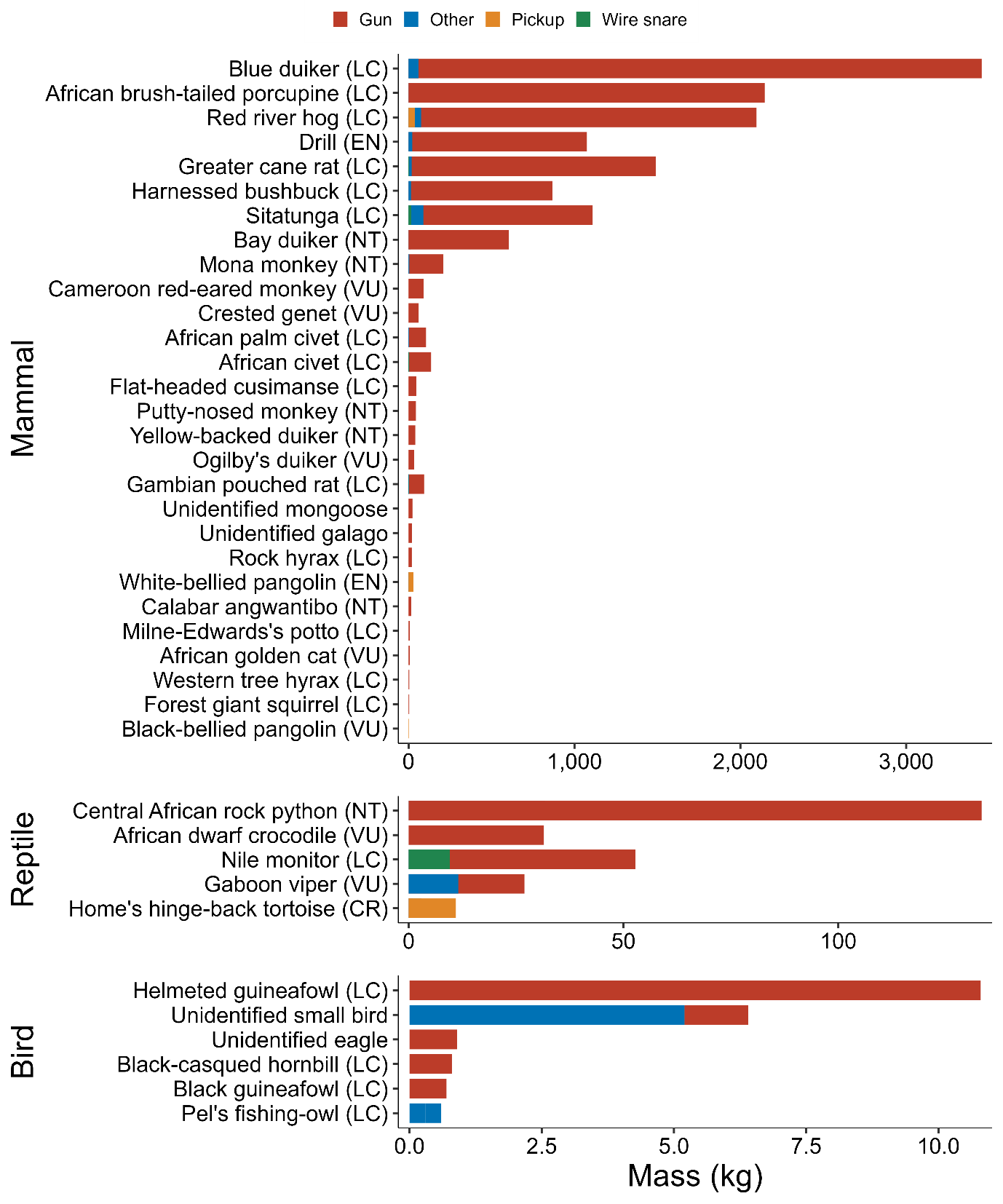


**Figure S9:** Total mass (in kilograms) of mammals, reptiles, and birds harvested across hunters. IUCN categories are written in brackets: LC = Least Concern, NT = Near Threatened, VU = Vulnerable, EN = Endangered, and CR = Critically Endangered. IUCN categories are in brackets: LC = Least Concern, NT = Near Threatened, VU = Vulnerable, EN = Endangered, and CR = Critically Endangered.


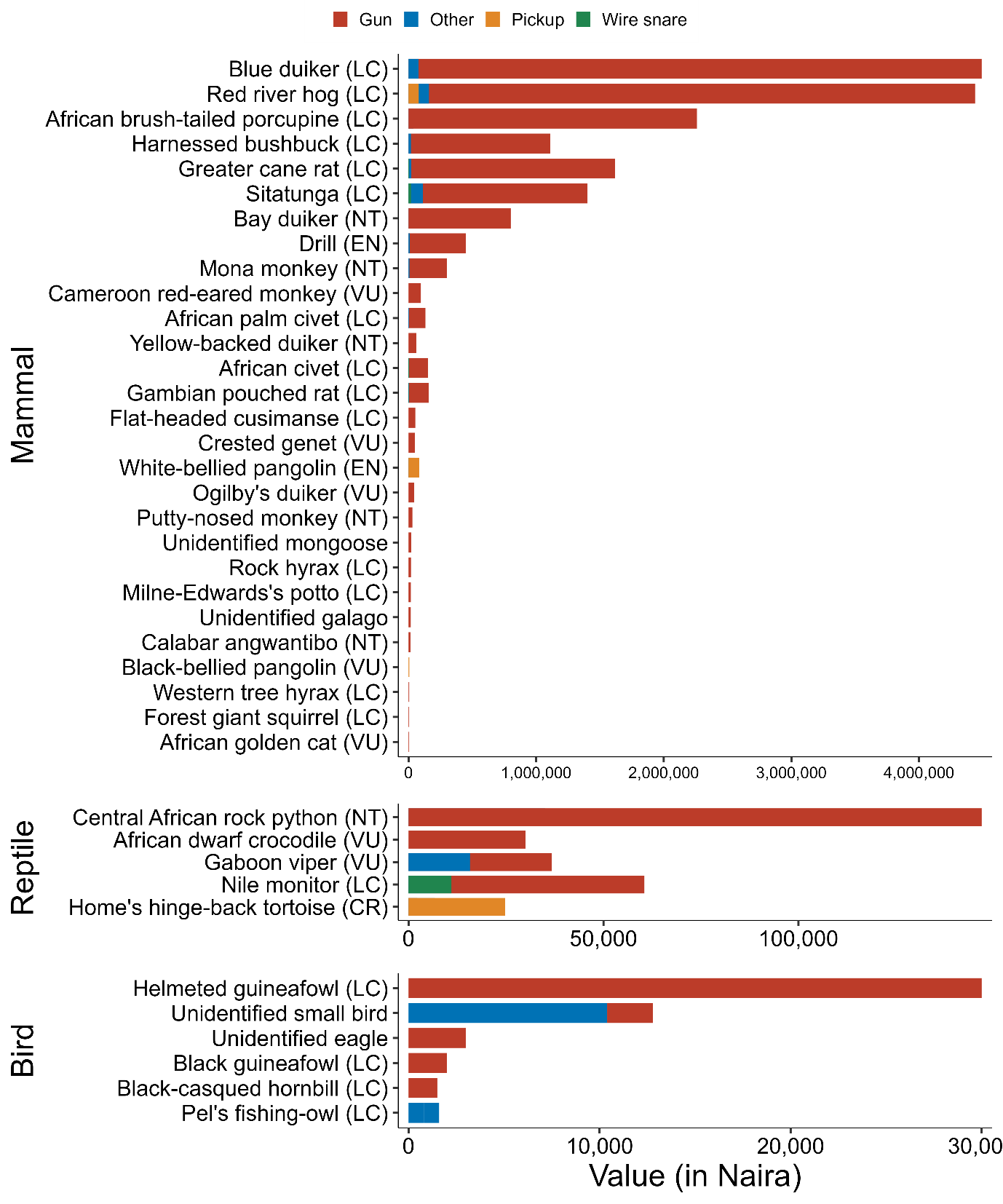


**Figure S10:** Estimated value (in Nigerian Naira) associated with wild meat offtake across hunters for mammals, reptiles, and birds. IUCN codes as in Figure S9.


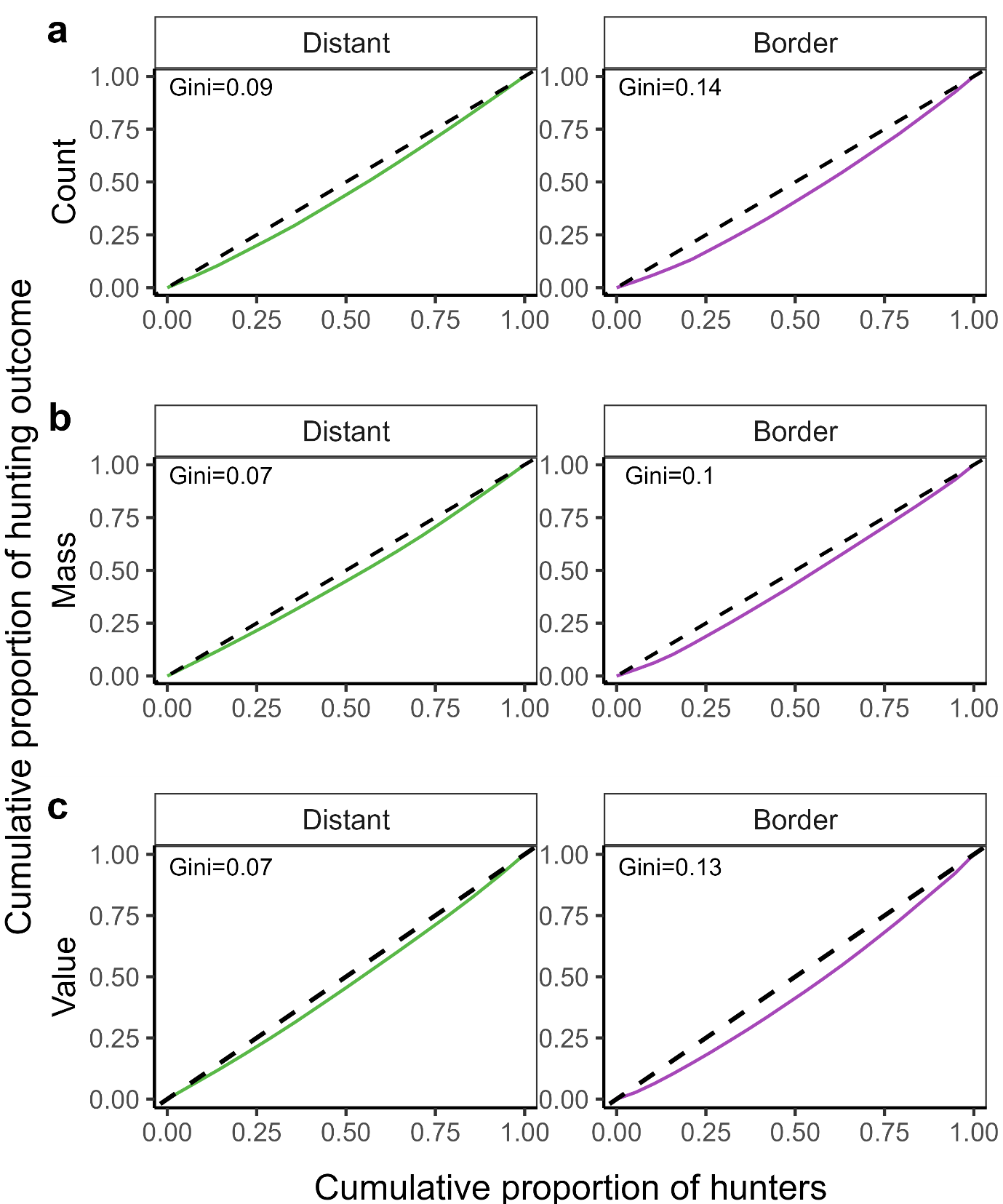


**Figure S11:** Lorenz curve for count (a), mass (b), and value (c). The dotted diagonal lines represent perfect equality for each hunting outcome. The continuous lines show the observed distribution of the corresponding hunting outcome. Gini coefficients for each outcome and community are shown on the plot. Coefficients and curves were estimated using R packages REAT (Wieland, 2019) and gglorenz (Chen & Cortina, 2020).


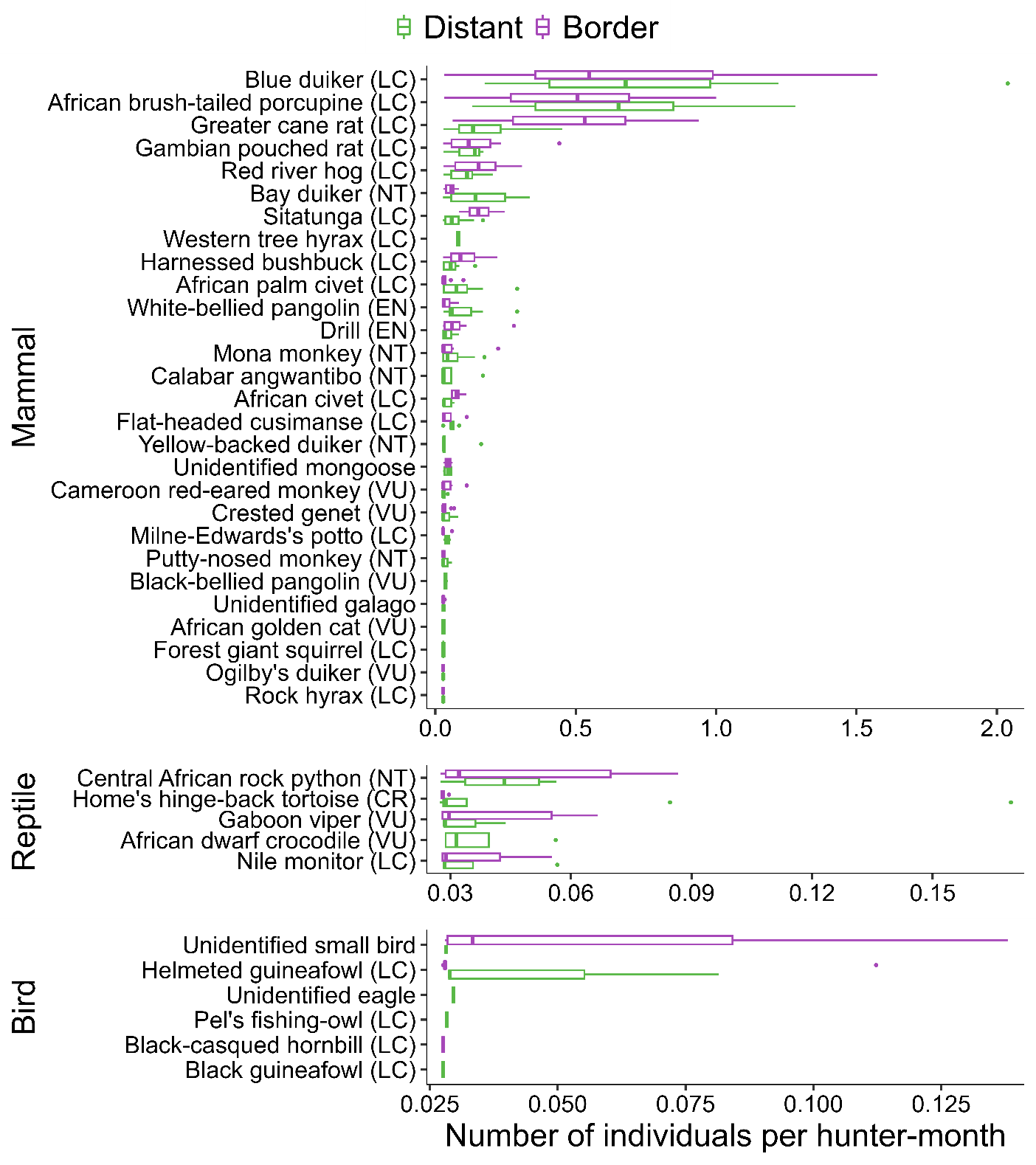


**Figure S12:** Boxplot summaries of the distribution of the number of individuals caught per hunter-month for each species of mammal, reptile, and bird. The central line shows the median, the box the interquartile range, lines the overall range, and any outliers are marked by dots. Values were adjusted by the hunting period of each hunter and the number of hunters in each community. Note the different scales for each taxon.

# **Appendix D: Appendix References**

Brashares, J. S., Golden, C. D., Weinbaum, K. Z., Barrett, C. B., & Okello, G. V. (2011). Economic and geographic drivers of wildlife consumption in rural Africa. *Proceedings of the National Academy of Sciences of the United States of America*, *108*(34), 13931–13936. https://doi.org/10.1073/pnas.1011526108

Brooks, M. E., Kristensen, K., Benthem, K. J. van, Magnusson, A., Berg, C. W., Nielsen, A., Skaug, H. J., Mächler, M., & Bolker, B. M. (2017). glmmTMB Balances Speed and Flexibility Among Packages for Zero-inflated Generalized Linear Mixed Modeling. *The R Journal*, *9*(2), 378–400. https://journal.r-project.org/archive/2017/RJ-2017-066/index.html

Chen, J., & Cortina, H. (2020). *gglorenz: Plotting Lorenz Curve with the Blessing of “ggplot2”* [Computer software].

Coad, L. M. (2008). *Bushmeat hunting in Gabon: Socio-economics and hunter behaviour* [Thesis, University of Cambridge]. https://doi.org/10/252091

Detoeuf, D., Wieland, M., & Wilkie, D. (2020). *Guide 2.0 to the Modified Basic Necessities Survey: Why and How to Conduct Digital-Based BNS in Conservation Landscapes*. https://doi.org/10.19121/2020.Report.38385

Godoy, R., Undurraga, E. A., Wilkie, D., Reyes-García, V., Huanca, T., Leonard, W. R., McDade, T., Tanner, S., Vadez, V., & Team, T. B. S. (2010). The effect of wealth and real income on wildlife consumption among native Amazonians in Bolivia: Estimates of annual trends with longitudinal household data (2002–2006). *Animal Conservation*, *13*(3), 265–274. https://doi.org/10.1111/j.1469-1795.2009.00330.x

Hartig, F. (2022). *DHARMa: Residual Diagnostics for Hierarchical (Multi-Level / Mixed) Regression Models.* [Computer software]. https://CRAN.R-project.org/package=DHARMa

Jones, S. C. Z., Papworth, S. K., St. John, F. A. V., Vickery, J. A., & Keane, A. M. (2020). Consequences of survey method for estimating hunters’ harvest rates. *Conservation Science and Practice*, *2*(12), e315. https://doi.org/10.1111/csp2.315

Koster, J., Mcelreath, R., Hill, K., Yu, D., Shepard, G., Vliet, N. V., Gurven, M., Kaplan, H., Trumble, B., Bird, R. B., Bird, D., Codding, B., Coad, L., Pacheco-Cobos, L., Winterhalder, B., Lupo, K., Schmitt, D., Sillitoe, P., Franzen, M., … Ross, C. (2019). *The Life History Of Human Foraging: Cross-Cultural And Individual Variation*. https://doi.org/10.1101/574483

Krieg, J. (2021). Influence of moon and clouds on night illumination in two different spectral ranges. *Scientific Reports*, *11*(1), Article 1. https://doi.org/10.1038/s41598-021-98060-2

Maidment, R. I., Grimes, D., Allan, R. P., Tarnavsky, E., Stringer, M., Hewison, T., Roebeling, R., & Black, E. (2014). The 30 year TAMSAT African Rainfall Climatology And Time series (TARCAT) data set. *Journal of Geophysical Research: Atmospheres*, *119*(18), 10,619-10,644. https://doi.org/10.1002/2014JD021927

Maidment, R. I., Grimes, D., Black, E., Tarnavsky, E., Young, M., Greatrex, H., Allan, R. P., Stein, T., Nkonde, E., Senkunda, S., & Alcántara, E. M. U. (2017). A new, long-term daily satellite-based rainfall dataset for operational monitoring in Africa. *Scientific Data*, *4*(1), Article 1. https://doi.org/10.1038/sdata.2017.63

Meirink, Jan Fokke, Karlsson, Karl-Göran, Solodovnik, Irina, Hüser, Imke, Benas, Nikos, Johansson, Erik, Håkansson, Nina, Stengel, Martin, Selbach, Nathalie, Marc, Schröder, & Hollmann, Rainer. (2022). *CLAAS-3: CM SAF CLoud property dAtAset using SEVIRI - Edition 3* (4.0, p. 78.3 TiB) [NetCDF-4]. Satellite Application Facility on Climate Monitoring (CM SAF). https://doi.org/10.5676/EUM_SAF_CM/CLAAS/V003

Ogutu, J. O., Piepho, H.-P., Dublin, H. T., Bhola, N., & Reid, R. S. (2008). Rainfall influences on ungulate population abundance in the Mara-Serengeti ecosystem. *The Journal of Animal Ecology*, *77*(4), 814–829. https://doi.org/10.1111/j.1365-2656.2008.01392.x

Prugh, L. R., & Golden, C. D. (2014). Does moonlight increase predation risk? Meta-analysis reveals divergent responses of nocturnal mammals to lunar cycles. *Journal of Animal Ecology*, *83*(2), 504–514. https://doi.org/10.1111/1365-2656.12148

SVS, N. (2021, November 18). *SVS: Moon Phase and Libration, 2022*. https://svs.gsfc.nasa.gov/4955

Tarnavsky, E., Grimes, D., Maidment, R., Black, E., Allan, R. P., Stringer, M., Chadwick, R., & Kayitakire, F. (2014). Extension of the TAMSAT Satellite-Based Rainfall Monitoring over Africa and from 1983 to Present. *Journal of Applied Meteorology and Climatology*, *53*(12), 2805–2822. https://doi.org/10.1175/JAMC-D-14-0016.1

Weisell, R., & Dop, M. C. (2012). The Adult Male Equivalent Concept and its Application to Household Consumption and Expenditures Surveys (HCES). *Food and Nutrition Bulletin*, *33*(3_suppl2), S157–S162. https://doi.org/10.1177/15648265120333S203

Wieland, T. (2019). REAT: A Regional Economic Analysis Toolbox for R. *REGION*, *7*(3), R1–R57. https://doi.org/10.18335/region.v6i3.267

Zuur, A. F., Hilbe, J. M., & Ieno, E. N. (2013). *A Beginner’s Guide to GLM and GLMM with R*. Highland Statistics Ltd. https://www.highstat.com/index.php/beginner-s-guide-to-glm-and-glmm
